# Thermo-flux: generation and analysis of thermodynamic-stoichiometric metabolic network models

**DOI:** 10.1038/s44320-026-00227-4

**Published:** 2026-07-03

**Authors:** Edward N Smith, Nathan Fargier, José Losa, Matthias Heinemann

**Affiliations:** 1 Department of Biology, University of Oxford, South Parks Road, Oxford, OX1 3RB UK; 2 Molecular Systems Biology, Groningen Biomolecular Sciences and Biotechnology Institute, University of Groningen, Nijenborgh 7, 9747 AG Groningen, The Netherlands

**Keywords:** Computational Biology, Metabolism

## Abstract

Metabolic modeling with stoichiometric models and flux balance analysis (FBA) has greatly advanced our understanding of metabolism. However, valid FBA predictions require mechanistically correct constraints. Thermodynamic constraints can increase the mechanistic foundations of stoichiometric models and reduce the solution space, but incorporating them has so far required cumbersome manual effort. To circumvent manual curation, we introduce ’Thermo-Flux’, a semi-automated Python package that converts stoichiometric models into comprehensive thermodynamic-stoichiometric models. ’Thermo-Flux’ enables (i) automated mass and charge balancing while considering physical and biochemical parameters, (ii) definition of transporter variants and Gibbs energies for transport processes, (iii) handling of metabolites with unknown structures or Gibbs energies, and (iv) integration of recent methods for determining Gibbs energies and their uncertainties. To guide users, we provide detailed instructions on how to use ’Thermo-Flux’ and include background information to facilitate appropriate modeling assumptions. We highlight the applicability of ’Thermo-Flux’ by converting 87 stoichiometric models from the BiGG database and demonstrate improved flux predictions for a genome-scale yeast model (iMM904). We expect ’Thermo-Flux’ to support fundamental and applied metabolic research.

## Synopsis

Thermo-Flux is a semi-automated Python pipeline that converts stoichiometric metabolic models into combined thermodynamic-stoichiometric models. Thermodynamic-stoichiometric models are mechanistically more accurate, and thus enable improved flux predictions.

- Thermo-Flux enables semi-automated conversion of stoichiometric models into combined thermodynamic-stoichiometric models.
- Thermo-Flux successfully processed 87 out of 107 BiGG models.
- Constraining a combined thermodynamic-stoichiometric model with metabolome data revealed incorrectly defined reaction directions.
- A combined thermodynamic-stoichiometric model achieved accurate predictions of overflow metabolism.

## Abstract (web summary)

Thermo-Flux is a semi-automated Python pipeline that converts stoichiometric metabolic models into combined thermodynamic-stoichiometric models. Thermodynamic-stoichiometric models are mechanistically more accurate, and thus enable improved flux predictions.

## Graphical abstract

**Figure:**
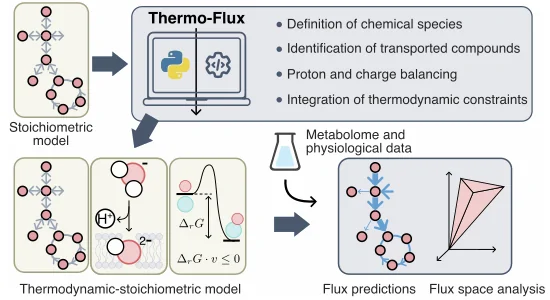

**Figure:**
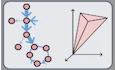

## Introduction

Metabolism is inherently complex and mathematical models can advance our understanding of this complexity. Genome-scale stoichiometric metabolic models combined with constraint-based approaches, most notably Flux Balance Analysis (FBA), have yielded valuable insights into metabolism (Orth et al, 2010; Ye et al, 2022), ranging from the identification of essential metabolic functions and genes (Carter et al, 2023; Weaver et al, 2014) to the discovery of metabolic engineering targets (Satanowski et al, 2025; Smith et al, 2025). However, FBA can still also produce incorrect flux predictions.

In addition to constraints on proteome allocation (Chen et al, 2021; Kerkhoven, 2022; Zare and Fleming, 2024) or molecular crowding [preprint: (Carlson et al, 2024; Vazquez and Oltvai, 2016)], thermodynamic constraints can improve the mechanistic foundation of stoichiometric models. For instance, by constraining reaction directions and thus reducing the feasible solution space (Henry et al, 2007; Krumholz and Libourel, 2017; Weilandt et al, 2021). Thermodynamic constraints also allow the integration of measured metabolite concentrations (Kümmel et al, 2006a,b; Weilandt et al, 2021). The value of thermodynamic constraints in stoichiometric models was demonstrated by the ability to reveal fundamental cellular mechanisms, such as thermodynamic bottlenecks or metabolite channeling (Gollub et al, 2021; Noor et al, 2014; Salvy and Hatzimanikatis, 2020). Finally, using comprehensive thermodynamic-stoichiometric models for *S. cerevisiae* and *E. coli*, we recently identified a possible limit in the cellular Gibbs dissipation rate (Niebel et al, 2019). Thus, adding thermodynamic constraints in stoichiometric models has been found to be powerful. However, the manual effort required to convert stoichiometric models into such comprehensive thermodynamic-stoichiometric models has hampered their widespread use.

To enable a broader application of thermodynamic constraints in metabolic modeling, automated tools are needed that reduce manual efforts during the development of such models. Tools such as multiTFA (Mahamkali et al, 2021), pyTFA and matTFA (Salvy et al, 2019), probabilistic thermodynamic analysis (PTA) (Gollub et al, 2021), ThermOptCobra (S and Bhatt, 2025) or ETGEM (Yang et al, 2021) address some associated challenges, such as improved estimation of thermodynamic parameters and integration of metabolomics data. However, they lack several important features, such as automated charge and proton balancing and proper curation of transporter variants, accurate modeling of Gibbs energies of transport processes, and guidance on how to handle the thermodynamics of atypical yet common reaction processes, such as biomass formation, electron transport chains or photochemical reactions.

To address these challenges, we introduce “Thermo-Flux”, a package designed to semi-automatically convert stoichiometric models into comprehensive thermodynamic-stoichiometric models. The key features of this package are: accurate charge and proton balancing based on physical and biochemical parameters of cellular compartments, automated definition of transporter variants and calculation of Gibbs reaction energies of transport processes, estimation of Gibbs energy of reactions with the component contribution method (Beber et al, 2022; Mahamkali et al, 2021; Noor et al, 2013), robust handling of metabolites with unknown structures or Gibbs formation energies, and the option to perform model regression to experimental data. In addition, we provide step-by-step instructions to guide users in the conversion of stoichiometric models into comprehensive thermodynamic-stoichiometric models. For each step, we describe the respective theoretical background and the assumptions made, illustrated by practical examples from a case study of the conversion of the yeast genome-scale metabolic network model (iMM904) (Mo et al, 2009). To demonstrate the applicability of “Thermo-Flux”, we converted 87 stoichiometric models from the BiGG database into thermodynamic-stoichiometric models, with minimal manual curation. Finally, we demonstrate the improved predictive capabilities of a fully parameterized thermodynamic-stoichiometric model. Overall, “Thermo-Flux” facilitates the development of comprehensive thermodynamic-stoichiometric models, which—through their stronger mechanistic foundation—will lead to better flux predictions and will thus support fundamental and applied metabolic research. Examples of the usage of “Thermo-Flux” are available in the online documentation of the Python package (https://thermoflux-rtd.readthedocs.io/en/latest/).

## Results

### Overview of the workflow

**Figure. 1:**
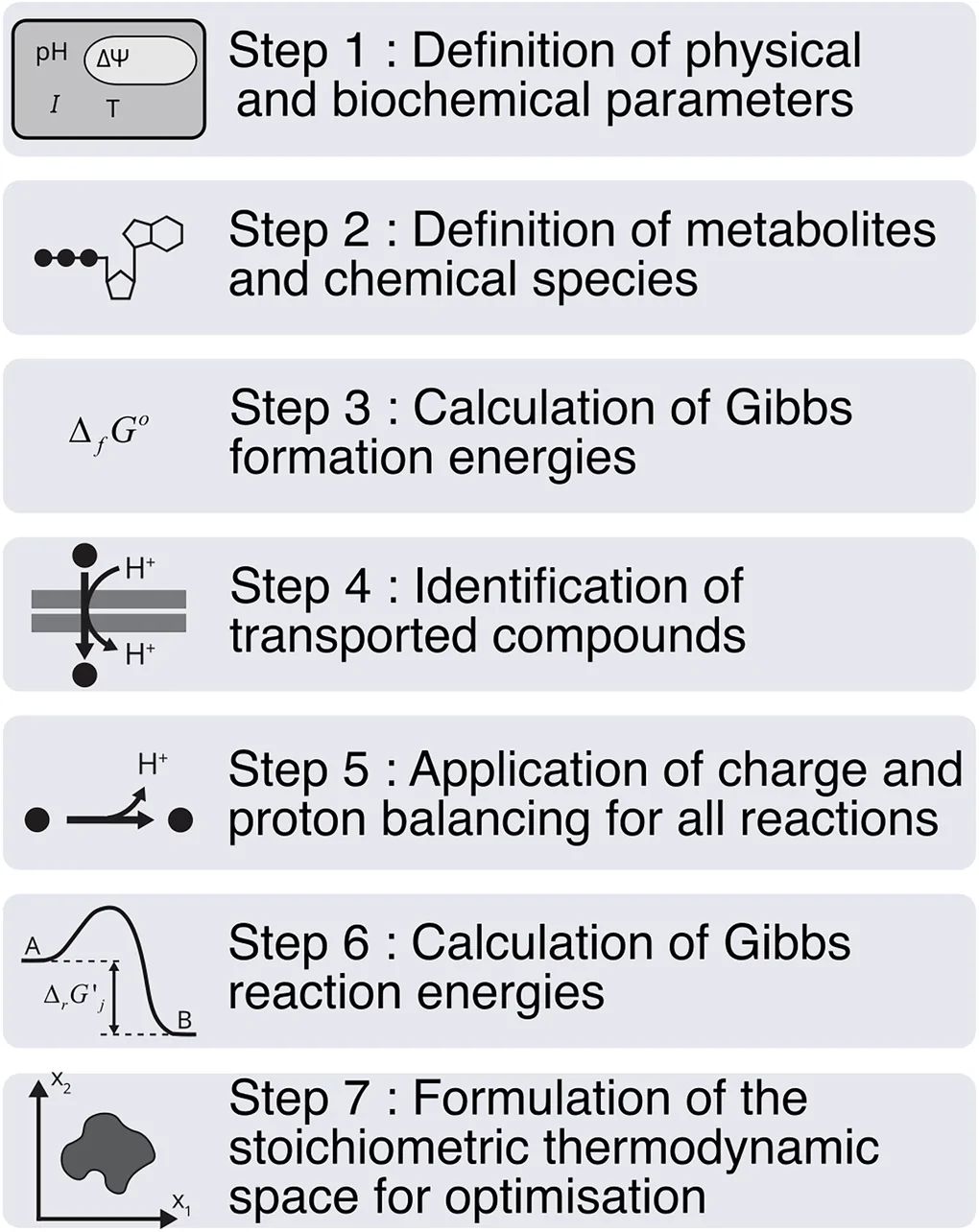
Overview of the different steps. Followed by “Thermo-Flux” to convert a stoichiometric model into a comprehensive thermodynamic-stoichiometric model.

The workflow of “Thermo-Flux” to convert stoichiometric models into thermodynamic-stoichiometric models is divided into the following steps (Fig. 1): (i) definition of physical and biochemical parameters (e.g., pH and ionic strength), (ii) definition of metabolites and their chemical species, (iii) calculation of metabolites’ Gibbs formation energies, (iv) identification of transported compounds, (v) application of pH-dependent charge and proton balancing, (vi) calculation of standard Gibbs energy of reactions including uncertainty, and (vii) formulation of the combined thermodynamic-stoichiometric solution space for constraints-based optimization. This last step also includes the possibility to fit model variables to experimental data, such as physiological data or metabolite concentrations. In the following, we provide instructions on how to perform each of these steps. In Appendix Table S1, we also provide code snippets that illustrate relevant steps of the conversion of the iMM904 model.

#### Step 1. Definition of physical and biochemical parameters

The thermodynamics of biochemical reactions and transport processes are influenced by physical and biochemical parameters. For instance, pH, pMg, and ionic strength determine the protonation and charge states of metabolites and their Gibbs formation energies. Membrane potential differences determine the Gibbs energy of transport reactions, and temperature influences all Gibbs reaction energies. These parameters must be defined in the following manner: while temperature (*T*) assumes a single value in the model, other parameters, such as pH, pMg, ionic strength (*I*), can take on different values in each of the subcellular compartments (cf. Table 1). In addition, membrane potential differences (*Δϕ*) are defined for pairs of compartments, i.e., the environments on either side of a membrane. Unless specified by the user, “Thermo-Flux” uses default parameters for all subcellular compartments, i.e., pH = 7.0, pMg = 3.0, ionic strength = 0.25 M, membrane potential difference = 0 mV between two compartments. However, users are advised to give preference to values reported for the specific organism and conditions, as these values are important for thermodynamic calculations. As a reference, we provide example values for these parameters for *E. coli*, *S. cerevisiae*, and *Arabidopsis* (Table EV1).

**Table 1:** Physical and biochemical parameters (and their units) used in the iMM904 case study.

Parameter	Unit	Example values
Potential hydrogen (pH)	Dimensionless	Cytosol:7, mitochondria:7.4, extracellular space:5
Potential magnesium (pMg)	Dimensionless	All compartments:14
Temperature (T)	Kelvin (K)	T:298 K
Ionic strength (I)	mol L^-1^ (M)	All compartments:0.25 M
Membrane potential difference (*ΔΦ*)	Volt (mV)	Plasma membrane:60 mV, mitochondrial membrane:160 mV

The code snippets used to set these parameters are in Appendix Table S1, snippet 1.1.

#### Step 2. Definition of metabolites and chemical species

**Figure. 2:**
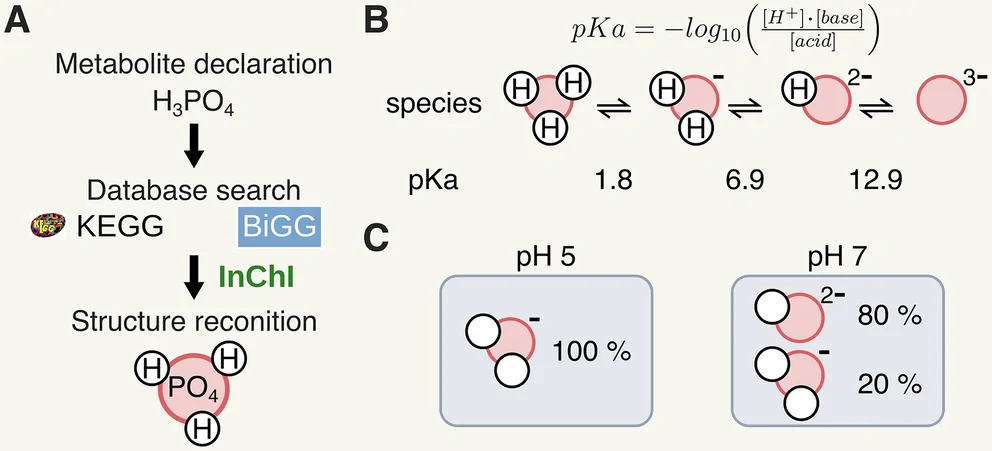
From metabolite definition to chemical species specification. (**A**) From the definition of a metabolite using one of the multiple database annotations, the metabolite structure is obtained from the respective database or explicitly defined with its InChi notation. (**B**) From *pK*_*a*_ calculation based on ChemAxon and the pH, the *eQuilibrator* package is then used to calculate the species distribution. (**C**) Proportions of each species of phosphate in pH 5 and in pH 7. The average protonation and charge states of phosphate vary in these two different conditions (2 protons and −1 charge at pH 5, 1.2 protons and −1.8 charge at pH 7).

To be able to add proton and charge balances to the model and to calculate the Gibbs energies for reactions, we must first identify (i) the chemical structures of the metabolites in each reaction, and (ii) the chemical species that compose each metabolite and the proportions between the different species at the given physical and biochemical parameters. To identify the structure of a metabolite, we use *eQuilibrator*, which stores the structures of many biochemical compounds. To access these structures in *eQuilibrator*, we need to connect a metabolite, as provided in a reaction of a stoichiometric model, to the respective compound in *eQuilibrator*. This connection can be achieved using various annotations that are typically provided in stoichiometric models. The annotations that can be used in “Thermo-Flux” range from structural notations (InChI or SMILES) to database identifiers, such as KEGG or BiGG (Fig. 2A). Once each metabolite is linked to its corresponding compound in *eQuilibrator*, it is possible to query its chemical structure, as well as its acid and magnesium dissociation constants, i.e., *K*_*a*_ and *K*_*Mg*_, calculated using ChemAxon.

In aqueous solutions, metabolites exist as a combination of multiple species differing in protonation and charge states. The proportion between these different species is defined by their acid dissociation constants and the physical and biochemical parameters (i.e., ionic strength and pH) (Fig. 2B). For example, inorganic phosphate (P_i_) can exist as species PO_4_^3−^, HPO_4_^2−^, H_2_PO_4_^−^, and H_3_PO_4_ (Fig. 2B). In principle, we would have to explicitly model all species of every metabolite and include balance equations for all of these species. However, as this would significantly increase the number of variables in the model (more reactions and more metabolites), we merge the different species into what is collectively referred to as a reactant (Alberty, 2003).

We can determine the average protonation and charge states of a reactant (i.e., a combination of species) using its species distribution. Once the proportions between the different species are known, the average protonation and charge states of the reactant can be calculated. Because these averages are non-integers, when properly balanced (see step 5), biochemical equations can involve non-integer stoichiometric coefficients. For example, at pH 7.0, P_i_ is composed of about 80% HPO_4_^2−^ and 20% H_2_PO_4_^-^ resulting in an average of 1.2 protons and an average charge of −1.8 for the reactant *P*_*i*_ (Fig. 2C). “Thermo-Flux” automatically calculates the species distributions whenever a metabolite is defined. The user can query the protonation and charge states of the reactants and can inspect the resulting species distribution estimated by “Thermo-Flux” using the code snippets 2.1 and 2.2, respectively (Appendix Table S1).

For metabolites whose structures cannot be encoded by InChI, such as organometallics (e.g., heme O) or metal ions, or for metabolites with unknown or ambiguous chemical structures, such as acyl carrier proteins, the protonation and charge states cannot be calculated by “Thermo-Flux”. In these cases, we need to rely on literature references to select one species with a specific protonation and charge state that represents the average protons and charge of these metabolites. For example, for iMM904, we manually defined the charge of the redox couple ferricytochrome/ferrocytochrome, using the code snippet 2.3 (Appendix Table S1).

Similarly to the equilibrium between species with different protonation states, an equilibrium can exist between species that form different complexes with Mg^2+^, with their proportions depending on their affinity to magnesium. With the same approach as described above, *K*_Mg_ values are used by *eQuilibrator* to estimate an average number of magnesium ions for a metabolite.

#### Step 3. Calculation of Gibbs formation energies

The next step is to determine the standard Gibbs formation energies (*Δ*_*f*_*G*^∘^) for all metabolites. The Gibbs formation energy describes the Gibbs energy change required to form one mole of the metabolite from its constituent elements in their standard state (i.e., pH 7, concentration of 1 M). A way of estimating *Δ*_*f*_*G*^∘^ is to use the component contribution method (CCM) (Noor et al, 2013). This method starts by decomposing compounds into constituting “components” (i.e., smaller metabolites or chemical groups) with known Gibbs formation energies. The standard Gibbs formation energy of the metabolite is obtained by summing the standard Gibbs formation energies of its constituent “components”.

Using the CCM, we can obtain standard Gibbs formation energies of reactants from *eQuilibrator*. However, as mentioned, metabolites are present as combinations of species, which differ by their protonation states (cf. step 2) and Gibbs formation energy. The Gibbs formation energy of each successive protonation state of a metabolite can be expressed relative to the previous, less protonated form as

1 $${\Delta }_{f}{G}^{\circ }({{{\rm{A}}}}_{{{\rm{m}}}})={\Delta }_{f}{G}^{\circ }({{{\rm{A}}}}_{{{\rm{m-1}}}})+RT\ln (10)\,p{K}_{a,m},\quad m=1,\ldots ,n$$

where *pK*_*a*,*m*_ is the acid dissociation constant for the protonation step from *A*_*m*−1_ to *A*_*m*_, where *A*_0_ would denote the fully deprotonated species.

Importantly, because the pH or ionic strength of a cell usually do not reflect standard conditions, correct thermodynamic calculations require that all Gibbs formation energies are corrected for the actual state of the cell. Specifically, a certain pH value is defined for a cell or compartment, which implies that the concentration of protons is set and remains constant. The Gibbs formation energies then need to be adjusted to this specified pH. In “Thermo-Flux”, these adjustments are achieved by combining a Legendre transformation (to correct for constant pH) with the extended Debye–Hückel equation (to correct for ionic strength) (Alberty, 2003), according to

2 $${\Delta }_{f}{G}_{l,i}^{{\prime}\circ }={\Delta }_{f}{G}_{l,i}^{\circ }-{N}_{l,i}^{H}RT\ln (10)\,{{\mbox{pH}}}\,-RT1.201{z}_{l,i}^{2}-{N}_{l,i}^{H}\frac{\sqrt{I}}{1+1.6\sqrt{I}}$$

where *N*^*H*^ is the average number of hydrogen atoms that can bind to the reactant in the species, *I* is the ionic strength and *z* the charge of the species *l*. The Gibbs formation energy of a metabolite is then the “Boltzmann-weighted sum” of the transformed Gibbs formation energies of the constituent species (Alberty, 2005):

3 $${\Delta }_{f}{G}^{{\prime}\circ }_{i}=-RT\,\ln {\sum}_{l}{e}^{-{\Delta }_{f}{G}_{l,i}^{{\prime}\circ}/RT}$$

Here, ${\Delta }_{f}{G}_{i}^{{\prime}\circ }$ is the transformed Gibbs formation energy of metabolite *i*, *R* is the universal gas constant, and *T* the temperature. The Boltzmann weight for a species *l* of metabolite *i*, $\exp (-{\Delta }_{f}{G}_{l,i}^{{\prime}\circ }/RT)$, is determined by its transformed Gibbs energy of formation, which depends on the specified pH (Eq. (2)) and its *pK*_*a*_ (Eq. (1)). The fraction of this species *α*_*l*,*i*_ under the specified conditions is obtained by normalizing its Boltzmann weight by the sum of the Boltzmann weights of all species ${\alpha }_{l,i}=\frac{\exp (-{\Delta }_{f}{G}_{l,i}^{{\prime}\circ }/RT)}{{\sum }_{l}\exp (-{\Delta }_{f}{G}_{l,i}^{{\prime}\circ }/RT)}$.

In principle, ${\Delta }_{f}{G}^{{\prime}\circ }$ values are condition-independent parameters. However, since the component contribution method is based on experimental data, it provides estimates of these parameters and their respective uncertainties. These uncertainties can be caused by errors in the measurements of equilibrium constants or systematic errors embedded in the assumptions made in the component contribution method (Noor et al, 2012, 2013). In “Thermo-Flux”, we take along these uncertainties in the developed models to avoid biasing the Gibbs energies towards a single set of parameter values that could contain errors.

After having defined the physical and biochemical parameters (cf. step 1) and the metabolites of the model (cf. step 2), we used the code snippet 3.1 (Appendix Table S1) to retrieve *Δ*_*f*_*G*° values and their corresponding uncertainties from *eQuilibrator*, and subsequently calculate the transformed ${\Delta }_{f}{G}^{{\prime}\circ }\,$ for all metabolites of the iMM904 model (Box 1).

#### Step 4. Identification of transported compounds

Transport reactions are processes that move metabolites across membranes. However, not all species of a reactant are necessarily transported, as transporter proteins can be specific to a particular species (Robinson and Kunji, 2006). In turn, the protonation state of the transported species and its charge will determine the Gibbs energy change of the transport process (cf. step 6) (Jol et al, 2010). Therefore, to correctly describe the thermodynamics of a transport process, it is important to know and define the specifically transported species. The identification of the transported species can be based on biochemical data, as obtained from transport assays, or can be inferred from transporter structures (Drew et al, 2021), if available.

**Figure. 3:**
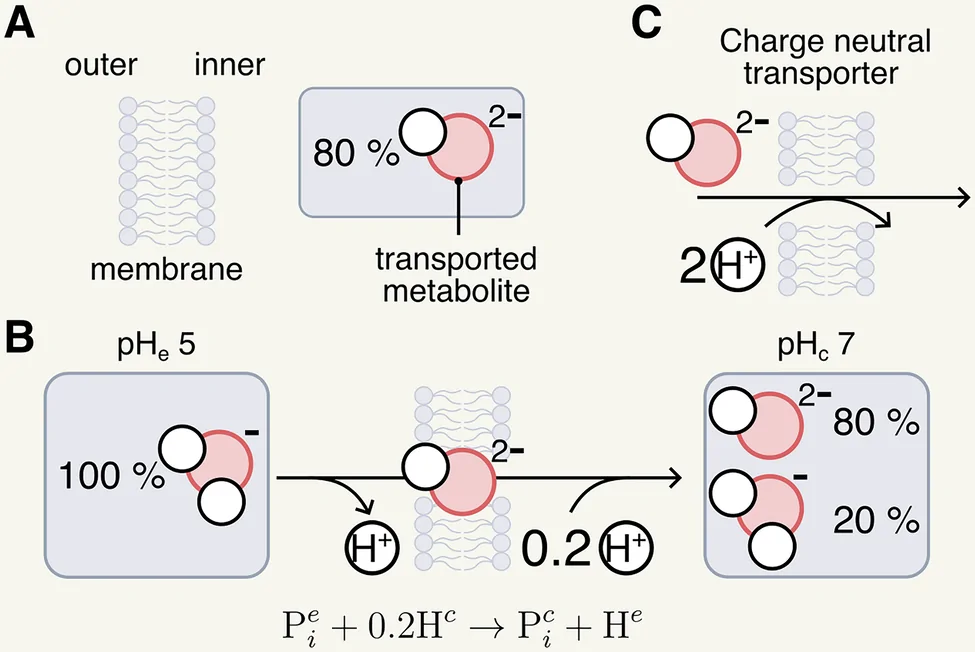
Identification of the transported compound. (**A**) In the inner compartment, “Thermo-Flux” identifies the major species of phosphate at pH 7; this species (HPO_4_^2−^) is assumed to be transported. (**B**) In the uniport process of phosphate transport, the major species in the inner compartment, HPO_4_^2−^, is transported from a compartment at pH 5 to a compartment at pH 7. At pH 5, the major species is, however, H_2_PO_4_^−^, which leaves one proton in the outer compartment when the transport as HPO_4_^2−^ occurs. As HPO_4_^2−^ crosses the membrane with one proton, 20% of the species gain one additional proton from the inner compartment. The final equation is thus ${\,{\mbox{P}}}_{i}^{e}+0.2{{\mbox{H}}}^{c}\to {{\mbox{P}}}_{i}^{c}+{{\mbox{H}}}^{e}$. Here, the superscript “e” and “c” refer to the compartments of the respective metabolite. (**C**) Proton symport (i.e., transport of a metabolite coupled with the transport of a proton) of phosphate is the charge-neutral transport of phosphate between an outer compartment at pH 5 and an inner compartment at pH 7.

Because such information is often not available, “Thermo-Flux” uses the assumption that the species with the highest abundance in the inner compartment is the one that is being transported. Specifically, the process through which “Thermo-Flux” determines the transported species, its protonation state and the net charge movement across the membrane is the following. First, for any given transport process, metabolites that occur on both sides of the reaction equation are identified as reactants whose species are transported across the membrane. Second, following our assumption, the species that has the highest abundance in the inner compartment is selected as the one that is transported. Third, we calculate the protonation state of this species and the amount of charge that crosses the membrane during its transport (Fig. 3A). For example, considering the transport of phosphate from the extracellular space (pH 5) to the cytosol (pH 7) and applying the steps mentioned above, we (i) identify that phosphate is the transported metabolite, (ii) determine that the transported species is HPO_4_^2-^, because it is the most abundant species in the inner compartment, and (iii) determine that this transport process moves a species that has one proton and two negative charges.

Once the transported species and its protonation and charge state is identified, the final stoichiometry of the transport process can be determined. Because the pH and thus the protonation state of the reactant may differ between compartments, protons can bind or release during transport (Fig. 3B). The equilibration of the transported species with the species distribution on each side of the membrane must be considered in the charge or proton balance (cf. step 5). “Thermo-Flux” calculates the protons that bind or release in each compartment based on the protonation state of the transported metabolite (cf. step 2) and the pH in each compartment. In the previously mentioned example of phosphate transport, “Thermo-Flux” compares the protonation state of phosphate in each compartment with the transported species and determines that, as the different species of phosphate equilibrate in the cytosol, one proton dissociates in the extracellular space and an additional 0.2 protons associate in the cytosol (Fig. 3B).

If the specifically transported species has been experimentally identified, “Thermo-Flux” offers the possibility to specify this particular species. “Thermo-Flux” also allows the inclusion of several different transporter variants. Each variant represents the transport of a different species of the same metabolite, differing in protonation state and in net charge movement, and therefore potentially enabling a different thermodynamically feasible transport direction. Transporter variants can be defined by coupling the transport of a metabolite to the transport of other metabolites such as protons or other ions, either in the same direction (symport) or in the opposite direction (antiport). For instance, variants can be added to include transport of a charge-neutral species of a metabolite at a given pH, by co-translocating protons together with the transported species and exactly balance the metabolite’s charge. Such a transport variant ensures that for every metabolite, a transport variant exists that does not translocate net charge and therefore avoids over-constraining the model. For example, the major species of phosphate in the cytosol at pH 7, HPO_4_^2−^, has two negative charges. Its transport from the extracellular space into the cytosol involves the movement of these negative charges. To add a charge-neutral transporter of phosphate from the extracellular space to the cytosol, a proton symport for phosphate transport (Fig. 3C) can be added to the model. In the iMM904 case study, we added a charge-neutral variant for cytosolic and mitochondrial phosphate transporters using the code snippet 4.1 (Appendix Table S1).

Some transport processes are more complex, and couple transport of a metabolite with chemical transformations, such as in ATP-powered proton pumps (Ambesi et al, 2000). In general, these reactions are automatically handled by “Thermo-Flux” and the transported species is correctly identified. However, in some cases, the transported species is ambiguous, as either the same metabolite does not appear on both sides of the membrane, or protons pumped across the membrane cannot be distinguished from those being taken up or released by chemical transformations. In these ambiguous cases, “Thermo-Flux” flags the reactions for manual curation. In the iMM904 case study, the transported compounds could be automatically defined for most reactions, except for (i) cross-membrane dehydrogenase reactions and for (ii) reactions involving chemical transformations coupled to cross-membrane proton exchange. For instance, we had to specify the number of transported protons in the cytosolic ATPase (ATPS). In this reaction, a proton is pumped through the membrane during the chemical reaction, which we specified using code snippet 4.2 (Appendix Table S1). For the reaction catalyzed by dolichyl phosphate mannosyltransferase (DOLPMTcer), in which a chemical transformation occurs simultaneously with the transport, we had to manually specify the transported metabolite (code snippet 4.3, Appendix Table S1).

#### Step 5. pH-dependent charge and proton balancing

Most stoichiometric models do not contain accurate proton and charge balances. This is because the protonation and charge states of reactants are most often either not consistently or accurately defined. However, charge and proton balances, being a function of the specific compartmental pH values, are important to accurately describe the biochemical thermodynamics of a cell. To implement accurate proton and charge balances, “Thermo-Flux” first determines the net change in protons for each reaction, using the protonation state of every metabolite of this reaction as calculated in step 2. To then balance the reaction, “Thermo-Flux” compensates for any excess proton by adding the equivalent amount of proton to the opposite side of the stoichiometric equation. For example, at pH 7 and ionic strength 250 mM, hydrolyzing one ATP molecule releases 0.7 protons (Table 2). In this case, “Thermo-Flux” balances the reaction by adding 0.7 protons to the right-hand side of the equation, which corrects charge imbalances that arise from differences in protonation states.

**Table 2:** Proton and charge balance of ATP hydrolysis at pH 7.

	ATP	+ H_2_O	→ ADP	+ P_i_	Net
Average protons	12.1	2.0	12.2	1.2	−0.7
Average charge	−3.9	0.0	−2.8	−1.8	−0.7

In metabolic networks, transport processes can involve charge movement or can be charge-neutral. Movement of charges between compartments must balance out to satisfy the steady-state assumption, ensuring constant membrane potentials. Therefore, we introduce a pseudo-metabolite “charge” to store this information in the stoichiometric matrix and to track net charge transfer across membranes. With this pseudo-metabolite representing positive charges and the steady-state constraint (*S* ⋅ *v* = 0), charge balance is enforced between compartments. To model the transport of a negative charge, we use the transport of a positive charge with opposite stoichiometry. As an example, in the uniport of phosphate from the outer compartment at pH 5 to the inner one at pH 7, the transported species is HPO_4_^2−^ and carries two negative charges. Thus, once charge-balanced, this transport process is described by the reaction ${\,{\mbox{P}}}_{i}^{e}+0.2{{\mbox{H}}}^{c}+2{{\mbox{charge}}}^{c}\to {{\mbox{P}}}_{i}^{c}+{{\mbox{H}}}^{e}+2{{\mbox{charge}}}^{e}$ (Fig. 3B), with the superscript referring to the compartments (c for cytosol, e for extracellular). Note that this pseudo-metabolite can also be used to represent any cation, such as Na^+^. To balance the reactions of the iMM904 model in terms of protons and charges, we used the code snippet 5.1 (Appendix Table S1).

Similar to protons, magnesium ions can also be balanced. If magnesium ions are transported, they must also be considered in the charge balance. However, magnesium binding affinity constants may not be available for all metabolites. Thus, if magnesium ions are balanced, we recommend leaving magnesium ion transport between compartments unconstrained to prevent over-constraining the model with potentially incomplete magnesium ion balances. This can be done by adding a transport process for magnesium ions between each compartment, and not enforcing the second law of thermodynamics for this transport process, such that this process remains fully reversible.

#### Step 6. Calculation of Gibbs energy of reactions

**Figure. 4:**
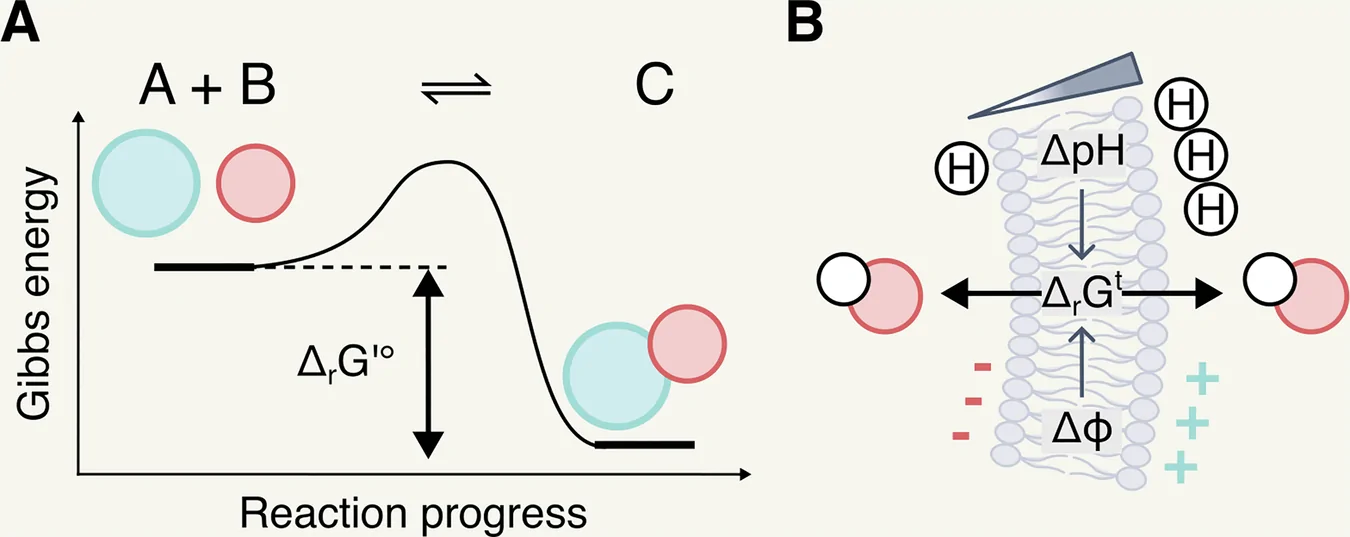
Calculation of Gibbs energy of reaction. (**A**) Under standard conditions, the Gibbs energy of reaction is the difference in Gibbs energy between the product C and the substrates (A + B). ${\Delta }_{r}G{{\prime} }^{\circ }$ is the standard Gibbs reaction energy transformed to the compartment’s physical and biochemical parameters (i.e., pH and ionic strength). (**B**) The transport term of the Gibbs reaction energy for a transport process also depends on the cell’s physical and biochemical parameters (i.e., differences in pH, *Δ*pH, and in membrane potentials, *ΔΦ*).

Next, we calculate the Gibbs energies of each reaction and transport process. With these Gibbs energies, we can constrain the direction of the reactions and transport processes by means of the second law of thermodynamics (Fig. 4A). In the following, we refer to both transport processes and chemical transformations as “reactions”. In a cell, the Gibbs energy of a reaction (*Δ*_*r*_*G*) differs from that under standard conditions. The Gibbs energy of reaction *j* is defined by:

4 $${\Delta }_{r}{G}_{j}={\Delta }_{r}{G}_{j}^{{\prime} \circ }+{\Delta }_{r}{G}_{j}^{c}+{\Delta }_{r}{G}_{j}^{t}$$

where ${\Delta }_{r}{G}_{j}^{{\prime} \circ }$ is the standard transformed Gibbs energy of the reaction, ${\Delta }_{r}{G}_{j}^{c}$ the Gibbs energy change due to the contribution of metabolite concentration, and ${\Delta }_{r}{G}_{j}^{t}$ the Gibbs energy change due to metabolite transport.

The first term, ${\Delta }_{r}{G}_{j}^{{\prime} \circ }$, is calculated from the difference between the standard transformed Gibbs formation energy of products and reactants and an additional correction term to account for uncertainty in Gibbs reaction energies:

5 $${\Delta }_{r}{G}^{{\prime }{ \circ }}_{j} =\sum {S}_{ij}{\Delta }_{f}{G}_{i}^{{\prime} \circ }\,+{\Delta }_{r}{G}_{j}^{\circ \,\,,error}$$

where *S*_*ij*_ is the stoichiometric coefficient of metabolite *i* in the reaction *j* and *Δ*_*r*_*G*^∘,*error*^ represents the uncertainty in Gibbs reaction energies. Because metabolites are shared among reactions, the uncertainty in *Δ*_*r*_*G*^∘^ must reflect the fact that the same metabolites participate in several reactions, meaning that these uncertainties are correlated. This correlation is captured using multivariate confidence intervals, computed with the square root covariance matrix of Gibbs reaction energies, *Q*, as returned by *eQuilibrator*, and an error vector *m*, which follows a normal distribution $m \sim {{\mathcal{N}}}(0,1)$, such that *Δ*_*r*_*G*^∘,*error*^ = *Q* ⋅ *m*. This formulation ensures that the *Δ*_*r*_*G*^∘,*error*^ values are internally consistent (Beber et al, 2022; Gollub et al, 2021; Mahamkali et al, 2021).

The second term of equation (4), ${\Delta }_{r}{G}_{j}^{c}$, accounts for the contribution of metabolite concentrations to the Gibbs energy of a reaction. This term is needed whenever metabolites are present at concentrations other than 1 M and can be calculated by,

6 $${\Delta }_{r}{G}_{j}^{c}=\,RT\,{\sum}_{i\,\ne \,{H}^{+}}{S}_{ij}\,\ln {c}_{i}$$

where *c*_*i*_ is the concentration of metabolite *i*. Note that concentrations can be used here instead of metabolite activities, because we use the extended Debye–Hückel theory in the calculation of Gibbs formation energies of metabolites (cf. step 2).

Finally, the third term of equation (4), ${\Delta }_{r}{G}_{j}^{t}$ is specific to transport processes and represents the Gibbs energy change due to the movement of charge or protons across the membrane (Fig. 4B). It is influenced by (i) the transport of species between compartments with different pH values and the release or binding of protons caused by protonation or de-protonation of the transported species, by (ii) the translocation of additional protons, e.g., by proton sym-/anti-porters or by proton pumps and (iii) the movement of charges between compartments with different membrane potentials. Taking these contributions into account, ${\Delta }_{r}{G}_{j}^{t}$, is defined by,

7 $${\Delta }_{r}{G}_{j}^{t}=-{N}_{H}RT\ln (10)\Delta \,{\mbox{pH}}-F{\mbox{q}}\,\Delta \Phi$$

where *N*_*H*_ is the number of free protons transported across the membrane, *Δ*pH is the pH difference across the membrane, q is the number of charges transported across the membrane (determined in step 5), *F* is the Faraday constant and *ΔΦ* is the membrane potential difference.

#### Step 7. Establishment of the thermodynamic-stoichiometric solution space

Initially, the flux solution space of a stoichiometric model is defined by typical FBA constraints, such as the steady-state mass balance equation:

8 S⋅v=0

and the flux bounds constraints, where each flux *v*_*j*_ of reaction *j* must be within its lower and upper bounds *lb*_*j*_ and *ub*_*j*_:

9 $${lb}_{j}\le {v}_{j}\le {ub}_{j}$$

Having determined the Gibbs reaction energies for all reactions in the model, including transport processes, thermodynamic constraints can be added to all these reactions. Importantly, the second law of thermodynamics constrains the direction of each reaction, such that only reactions with negative Gibbs reaction energy are allowed to proceed, according to:

10 $${\Delta }_{r}G\cdot v < 0$$

**Figure. 5:**
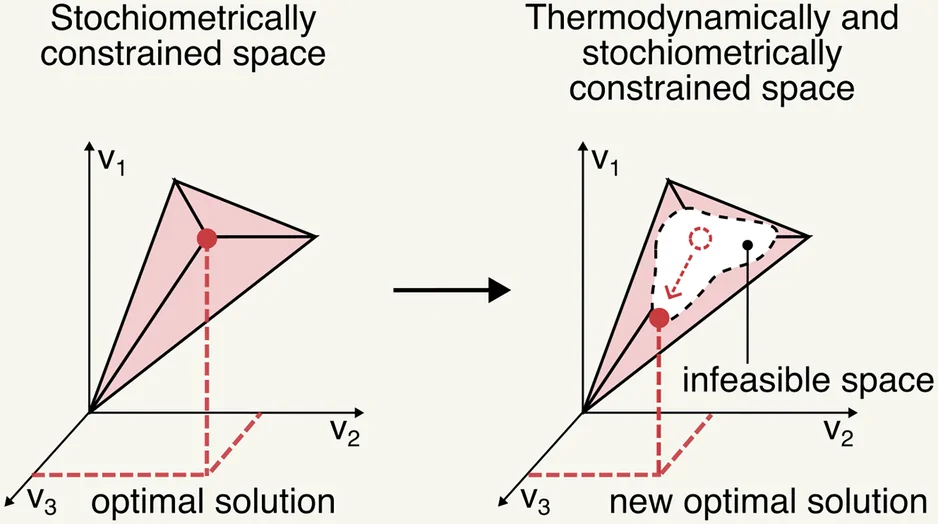
Illustration of the thermodynamic-stoichiometric solution space. The addition of the second law to the stoichiometrically constrained solution space removes thermodynamically infeasible combinations of reactions, turning the flux cone into a non-convex space and potentially altering the optimal solution.

These “second-law” constraints prevent thermodynamically infeasible combinations of reaction fluxes. Because both *Δ*_*r*_*G* and the fluxes *v* are variables of the optimizations, the second-law constraint (equation (10)) is bilinear and renders the solution space for the steady-state fluxes non-convex (Fig. 5). This non-convexity increases computational cost and runtime (cf. Table EV2), particularly on models with reversible reactions, as the number of possible direction combinations scales exponentially with the number of reactions. Runtime can be reduced by restricting the optimization to a subset of reactions, for instance by determining flux-coupled reactions (Erdrich et al, 2015) beforehand.

Beyond these constraints, “Thermo-Flux” also allows addition of a constraint on the cellular Gibbs energy dissipation rate, representing the sum of the products of the Gibbs reaction energy and the corresponding reaction flux, taken over all exchange reactions (see Appendix methods 5.2). Using an upper limit in the cellular Gibbs energy dissipation rate has previously been shown to improve flux predictions, particularly those associated with overflow metabolism (Niebel et al, 2019).

We used code snippet 7.1 (Appendix Table S1) to setup an FBA optimization with thermodynamic constraints for the iMM904 model. A complete overview of all model variables and constraints is provided in Table EV3 and additional considerations are discussed in Box 2. “Thermo-Flux” allows for the selection of various objective functions for the optimizations, such as maximizing growth rate (i.e., biomass production), energy production or minimal ATP or redox potential consumption or fitting the model variables to experimental data through regression analysis (Box 3). (Henry et al, 2007; Weilandt et al, 2021). Once the objective has been selected, the code snippet 7.2 (Appendix Table S1) is used to optimize the model using the Gurobi solver (Gurobi Optimization, LLC, 2023).

Box 1: Additional considerations for Gibbs formation energy calculations **Metabolites with unknown or non-decomposable structure**

Gibbs formation energies cannot be estimated for metabolites with an unknown structure or with a structure that cannot be decomposed by the component contribution method. For such metabolites, we assign a Gibbs formation energy of 0 kJ/mol, in combination with a large uncertainty of ±3000 kJ/mol. This wide uncertainty range reflects the fact that the metabolite’s ${\Delta }_{f}{G}^{{\prime} \circ }$ could lie anywhere within this range, potentially allowing the Gibbs energy of the reaction involving this metabolite to take any positive or negative value. To control how strongly metabolites with unknown or non-decomposable structure influence the constraining effect on reaction direction, “Thermo-Flux” allows the user to adjust this default uncertainty in ${\Delta }_{f}{G}^{{\prime} \circ }$.

**Redox carriers**

For protein-based or membrane-bound redox carriers, such as cytochrome C, the ${\Delta }_{f}{G}^{{\prime} \circ }$ can be calculated from experimentally determined midpoint potentials using the Nernst equation for a redox half reaction,

$${\Delta }_{f}{G}^{{\prime} \circ }=-\frac{1}{2}nF\Delta E$$

where *n* is the number of electrons transferred, *F* is the Faraday constant and *ΔE* is the redox reaction’s midpoint potential difference (in volt V).

**Photons**

Photosystems of plants and other organisms relying on photosynthesis are an important part of their metabolism, as these organisms transfer the energy from photons to redox cofactors. Therefore, in a combined thermodynamic-stoichiometric model of such an organism, the Gibbs formation energy of photons must be defined to accurately describe the thermodynamics of the photosynthesis reactions. Published stoichiometric models that include photons as metabolites exist for cyanobacteria (e.g., iJN678 and iSynCJ816 in BiGG) and algae (e.g., iLB1027_lipid in BiGG). The Gibbs formation energy of a photon is related to its wavelength *λ* by:

$${\Delta }_{f}{G}^{\circ }=\frac{hc}{\lambda },$$

with *h* being Planck’s constant and *c* the speed of light in the growth medium.

**Biomass**

In stoichiometric models, biomass is also considered as a metabolite. To comprehensively describe the thermodynamics of the whole metabolic network, including biomass formation, a Gibbs formation energy must also be specified for biomass. Values for Gibbs formation energies of biomass have been estimated based on the elemental composition of biomass and empirical formulas (Battley, 1999; Popovic, 2019). These empirical estimates must also be transformed based on the number of hydrogen atoms in the biomass empirical formula (Eq. (2)). Transformation of the biomass’s Gibbs formation energy is important when applying a constraint on the rate of the cellular Gibbs energy dissipation, as this Gibbs formation energy contributes to the total Gibbs energy dissipation (Niebel et al, 2019).

In “Thermo-Flux”, users can manually define Gibbs formation energies and associated uncertainties for the special metabolites mentioned in this box.

Box 2: Additional considerations for the establishment of the thermodynamic/stoichiometric constrained solution space **Metabolite concentration bounds**

Typically, intracellular metabolite concentrations vary between 0.1 μM and 10 mM (Bennett et al, 2009; Kümmel et al, 2006a), and this range is chosen as the default concentration range. For metabolites with concentration measurements reported in the literature, the default bounds can be adjusted to describe the minimal and maximal reported values.

**Certain metabolite concentrations can be expressed as ratios**

While for most metabolite concentration bounds are required for each metabolite, for redox cofactors, ratios of concentrations could be sufficient to constrain the thermodynamics of the reaction. Using ratio is advantageous for redox cofactors, because while their concentrations are difficult to measure, their ratios are known, even in different compartments (Bekers et al, 2015; Canelas et al, 2008). To apply a constraint on the redox cofactor ratio, we set one cofactor to 1 M. By doing so, its contribution to the reaction energy equation becomes zero (since ln(1) = 0), reducing the two concentration variables to the concentration ratio of the cofactors. For example, for the NADH/NAD^+^ pair, fixing the concentration of NAD^+^ to 1 M ensures that the concentration of NADH directly represents the NADH/NAD^+^ concentrations ratio in all thermodynamic calculations. However, it is important to note that this approach can only be applied for metabolites that do not contain de novo synthesis reaction or salvage pathways in the model (such as Ferrocytochrome/Ferricytochrome in the iMM904 model), because the concentration of the synthesized or salvaged metabolite would appear individually in such reactions.

**Compartment-specific metabolite concentrations**

The experimental determination of metabolite concentrations as they are present in subcellular compartments is experimentally challenging (Fürtauer et al, 2016; Klie, 2011). In contrast, whole-cell concentrations are readily available from metabolomics studies. Importantly, whole-cell concentration data can be expressed in terms of compartmental concentrations if the relative compartmental volumes are known. The whole-cell concentration of a metabolite is a weighted average of the concentration in the different subcellular compartments:

$${[A]}_{total}={[A]}_{1}{f}_{1}+\cdots +{[A]}_{n}{f}_{n}$$

where [*A*]_total_ is the concentration of a metabolite in the whole cell, [*A*]_*n*_ is the concentration of the metabolite in compartment *n* and *f*_*n*_ is the fractional volume of compartment *n*. Whole-cell concentration measurements can then be used as additional bounds in FBA optimizations (cf. step 7) or regressions, if the fractional volumes of compartments are known.

**Metabolites for which concentration are ignored**

The concentration of the pseudo-metabolite “charge” and of other pseudo-metabolites (e.g. biomass) are ignored when calculating the Gibbs energy term accounting for the contribution of metabolite concentrations, *Δ*_*r*_*G*^*c*^. The concentration of protons is also ignored in this term, as protons are already considered in the transformed Gibbs energies of formation (cf. step 3).

**Reactions where the second law of thermodynamics is not applied**

The second-law constraint is not applied for exchange reactions, where metabolites are exchanged across the system boundary. Importantly, the second-law constraint is also not applied to the biomass synthesis reaction, and to the transport of water between compartments, which is assumed to be reversible. To ignore the second-law constraint for a reaction, the code snippet B2 can be used (Appendix Table S1).

### Assessment of the workflow

**Figure. 6:**
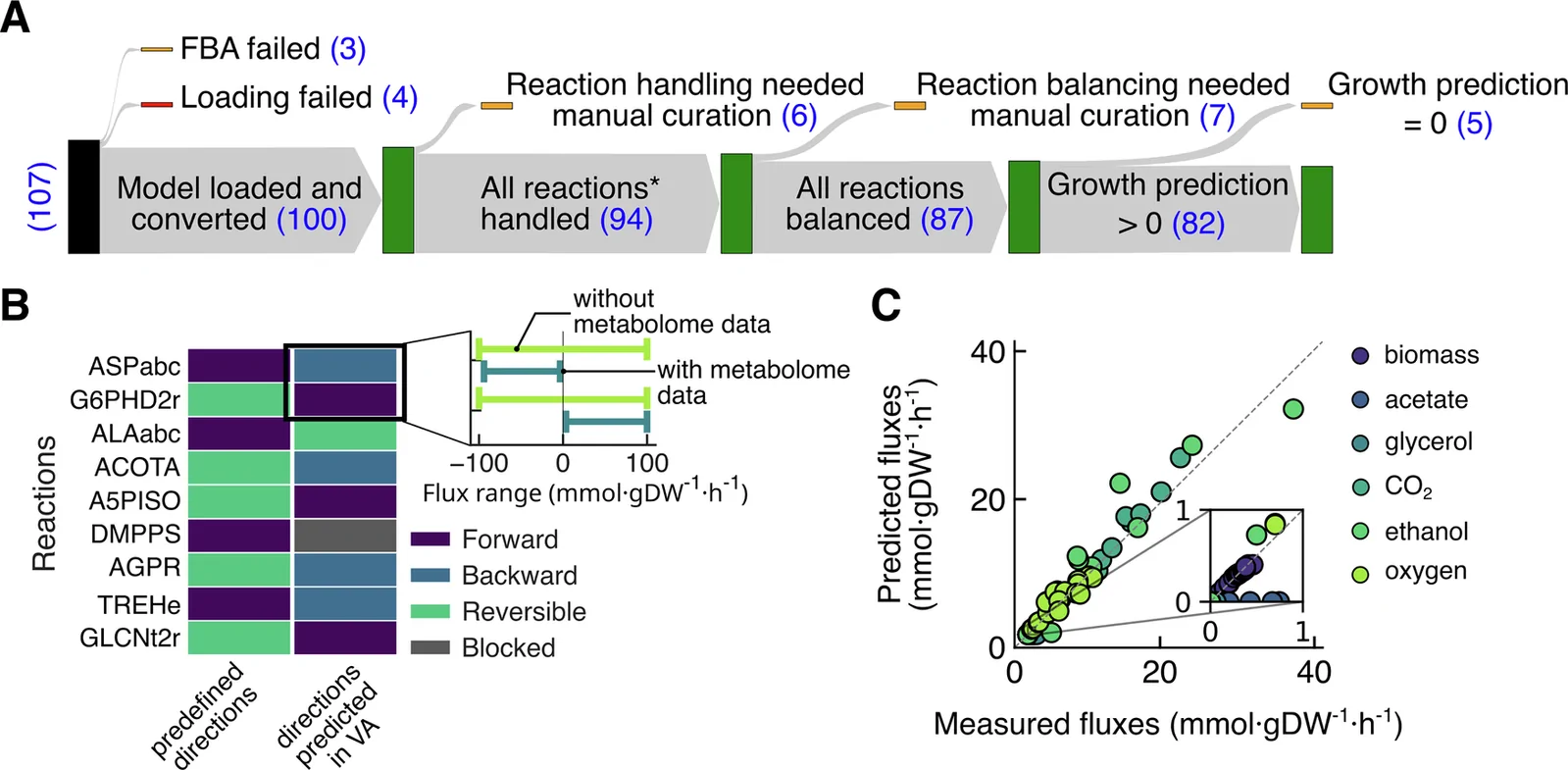
Conversion of stoichiometric models into combined thermodynamic-stoichiometric ones and flux space analysis using “Thermo-Flux”. (**A**) Application of the “Thermo-Flux” workflow to all 107 models from the BiGG database, with the number of models that passed each step of the workflow. (**B**) Comparison of reaction directions predefined in the iJR904 model with reaction directions predicted in variability analyses (VA) using a combined thermodynamic-stoichiometric model constrained with metabolome and physiological data. The color of the heatmap’s cells indicates the direction of each reaction shown: forward means the reaction can only carry flux in the forward direction, backward means it can only carry flux in the reverse direction, reversible means the reaction can carry flux in both directions, and blocked means the reaction cannot carry any flux. The inset plot zooms into the ASPabc and G6PHD2r reactions and shows the difference in flux ranges when performing variability analyses with and without metabolome data as bounds on measured metabolite concentrations. (**C**) Predictions of extracellular fluxes using the iMM904 model converted with “Thermo-Flux”, which incorporates thermodynamic constraints, predefined reaction directions and an upper limit on the cellular Gibbs energy dissipation rate. For each condition, we constrained the glucose uptake rate to values measured in the experiment and maximized the growth rate. The experimental data used for comparison with the model predictions were compiled from several published datasets, including (i) *S. cerevisiae* CEN.PK strain grown in glucose-limited chemostat cultures at different dilution rates (Canelas et al, 2011), (ii) DS2891X strain grown in glucose-limited chemostat cultures at different dilution rates and in one batch culture (Van Hoek et al, 1998), and (iii) data from a batch-cultured glucose-grown CEN.PK strain (Schmidt et al, 2014).

Having developed the “Thermo-Flux” workflow for the semi-automated conversion of stoichiometric models into combined thermodynamic-stoichiometric models, we assessed the workflow’s capacity to do so. To this end, we obtained all 107 stoichiometric models, reflecting various organisms, from the BiGG database (Norsigian et al, 2020) and ran them through the workflow. “Thermo-Flux” automatically performed the conversion of 87 models (77% of all available models). Among the 20 models that could not be automatically processed, we identified three main issues (Fig. 6A). First, seven models were already incorrectly formulated in the BiGG database. Specifically, four models were missing biomass equations or had incomplete model annotations (e.g., reactions missing compartment assignments), and three yielded infeasible FBA optimizations, based on the original stoichiometry alone. Then, six other models had transport or biomass reactions that could not be handled automatically by “Thermo-Flux” because they involved more than three compartments. Currently, “Thermo-Flux” only handles reactions involving up to three compartments, as dealing with a larger number poses challenges in identifying the transported metabolites (see Appendix methods 5.3). Finally, in seven additional models, automatic balancing failed because of reactions that involved a chemical transformation during a transport process. For example, transport reactions via the phosphotransferase system could not be balanced automatically in certain *E. coli* models. Automatic balancing also failed when models contain metabolites with undefined or ambiguous structure or formula, which happened mostly for specific redox cofactors, such as ferricytochrome or electron-transferring flavoproteins. Overall, the high success rate shows that “Thermo-Flux” can facilitate the automated conversion of stoichiometric models into combined thermodynamic-stoichiometric ones.

We then investigated how many of the 87 converted models would allow for biomass synthesis when reaction directions are constrained by the second law of thermodynamics. For this, we added thermodynamic constraints (cf. step 7), default physical and biochemical parameters (cf. step 1), and applied broad ranges of metabolite concentrations (cf. Box 2), and defined the objective function as maximization of growth rate. We found that 82 models displayed growth (i.e., a growth rate above zero), showing that the addition of thermodynamic constraints did not prevent essential reactions from occurring in the direction necessary for biomass formation. On the other hand, five models, which had displayed growth rates above zero prior to the addition of these constraints, no longer displayed growth. We identified two main causes for such issues. First, we found that in some models the proton balance could not close, potentially caused by incorrect reaction stoichiometries in the transport processes. This issue requires manual curation of transport processes, as demonstrated in the iMM904 case study, to ensure that the movement of protons is modeled accurately and that the resulting Gibbs reaction energies of the transport processes are accurate. Second, certain downloaded models contained biomass reactions with stoichiometric coefficients in the order of thousands (e.g., 35,115 ATP per biomass produced in iSB619), indicating that the biomass equation had not been scaled to produce 1 g of biomass. Such large stoichiometric coefficients scale *Δ*_*r*_*G*^∘^ values proportionally, yielding values so extreme that no feasible combination of metabolite concentrations and Gibbs energy uncertainties could satisfy the thermodynamic constraints, rendering the optimization infeasible. Overall, most models generated by “Thermo-Flux” could be used to predict fluxes using FBA with thermodynamic constraints, with failures often due to errors in the stoichiometry of the initial model.

Stoichiometric models commonly use predefined reaction directions, which are often defined ad hoc and reflect directions as they occur under standard conditions, such as growth on glucose. However, the accuracy of such assumptions on reaction directions is rarely tested. Therefore, we investigated the extent to which these predefined directions are correct by examining whether they would be consistent with reaction directions predicted by a mechanistically more correct model built with “Thermo-Flux”. To this end, we converted an *E. coli* stoichiometric model (based on the iJR904 BiGG reconstruction) with 623 reactions and 454 metabolites into a combined thermodynamic-stoichiometric model. Then, we constrained the combined thermodynamic-stoichiometric model using physiological data (Vila Chã Losa, 2024) for a glucose-grown culture and performed flux variability analyses with thermodynamic constraints. Here, we found that four predefined directions were incorrect compared to reaction directions as obtained from these variability analyses (Fig. 6B). When additionally constraining the model with metabolome data (Kochanowski et al, 2017), we found two more incorrectly predefined directions. Furthermore, incorporating metabolome data narrowed flux ranges, with 1.1% of reactions having an incorrect predefined direction and 8.1% becoming more constrained. demonstrating that metabolite concentration bounds additionally constrain reactions towards a specific direction (Fig. 6B). These results show that predefined reaction directions can include errors with potentially important consequences, and that integrating thermodynamic constraints with physiological and metabolome data provides a way to both verify assumptions on reaction directionality and further constrain flux ranges.

**Figure EV1:**
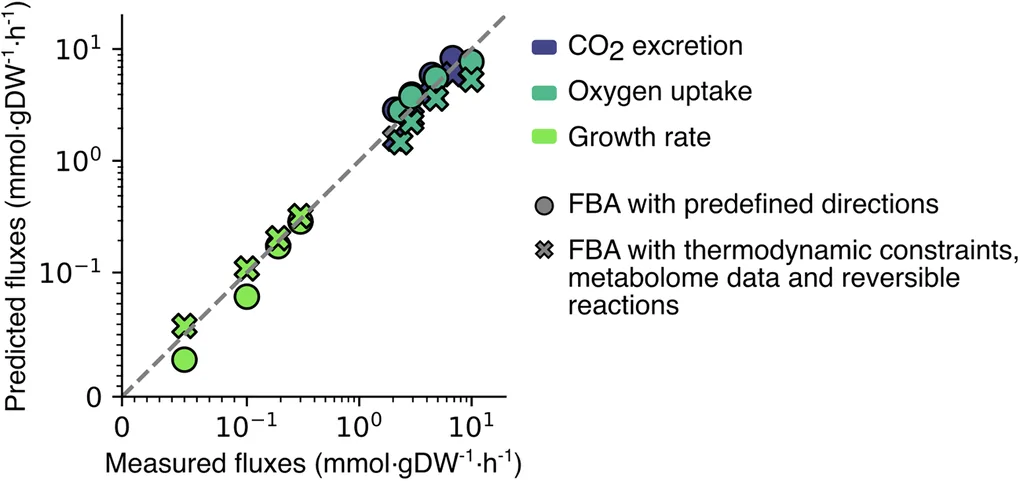
Comparison of predicted and experimental extracellular fluxes in *E. coli* at four low-glucose uptake rates. The fluxes were predicted using either (i) FBA with the iJR904 stoichiometric model with its predefined reaction directions, or (ii) FBA with the *E. coli* model built with “Thermo-Flux”, which incorporates thermodynamic constraints and metabolome data as bounds on measured metabolite concentrations, but fully reversible reactions. With the iJR904 model, loopless FBA and pFBA predicted the same results as standard FBA in terms of exchange fluxes, these predictions are not included in this figure. All FBA optimizations maximized biomass production. The experimental values were measured in chemostat cultures (Vemuri et al, 2007) and were measured only for CO_2_ excretion, O_2_ uptake and growth rate.

Next, we asked how predictions with thermodynamic constraints and metabolome data would compare to predictions with a stoichiometric model with predefined reaction directions. To do so, we constrained the glucose uptake rate to values measured in a chemostat experiment (Vemuri et al, 2007) and performed both standard FBA with a stoichiometric model with predefined directions, and FBA with thermodynamic constraints and reversible reactions. We focused on low-glucose uptake conditions, which do not exhibit overflow metabolism, because such phenotypes cannot be predicted without additional constraints such as an upper limit in the cellular Gibbs dissipation rate, a constraint on proteome allocation or on membrane crowding (de Groot et al, 2020). Here, FBA with thermodynamic constraints and reversible reactions predicted extracellular fluxes with an accuracy comparable to standard FBA with a stoichiometric model with predefined reaction directions (Fig. EV1). Thus, for low-glucose uptake conditions, thermodynamic constraints can be used to predict cellular physiology instead of relying on ad hoc assumptions on reaction directions, which, as seen above, may not always hold.

Finally, using a combined thermodynamic-stoichiometric model enables the use of an additional non-linear constraint to predict overflow metabolism, i.e., an upper limit on the cellular Gibbs dissipation rate (de Groot et al, 2020; Niebel et al, 2019). In contrast, FBA-derived approaches relying solely on stoichiometric constraints do not allow for the use of such additional constraints because they are not thermodynamically consistent and lack thermodynamic parameters. Thus, we asked whether flux predictions obtained from a model converted with “Thermo-Flux”, which incorporates thermodynamic constraints, predefined reaction directions and the additional dissipation rate constraint, would quantitatively agree with experimental data obtained under growth conditions that exhibit overflow metabolism. Here, using the iMM904 yeast model curated in our case study, we found that predictions of extracellular fluxes with different glucose uptake rates were in good agreement with experimental data, including overflow metabolism fluxes (Fig. 6C). These results show that models built with “Thermo-Flux” are capable of predicting complex phenotypes such as overflow metabolism, which remains out of reach for FBA-derived approaches relying solely on stoichiometric constraints.

Box 3: Regression—fitting models to experimental data Through a regression analysis, models can be fit to available data (e.g., metabolite concentrations and/or extracellular fluxes), enabling the inference of non-measured parameters (e.g., *Δ*_*r*_*G*^∘,error^). Furthermore, if implemented in the model, the cellular Gibbs energy dissipation rate can also be inferred from regression to experimental data (Niebel et al, 2019). In “Thermo-Flux”, the code snippet B3 (Appendix Table S1) can be used to add variables (e.g., whole-cell concentrations) to the previously constructed optimization problem with thermodynamic and stoichiometric constraints.

Then, the objective is to minimize the difference between predicted and experimental values of metabolite concentrations, extracellular fluxes, or both.. These regressions can be performed simultaneously on data from multiple experiments, such as those obtained from cells grown on different substrates. Such simultaneous regressions could help determine more accurate values of *Δ*_*r*_*G*^∘,error^, which would be consistent between datasets. There is a strong need for such improved *Δ*_*r*_*G*^∘,error^ estimates, particularly for reactions in which the component contribution method only provides values with high uncertainty.

## Discussion

To facilitate the use of thermodynamic constraints in metabolic modeling, we developed the “Thermo-Flux” workflow to convert stoichiometric metabolic network models into combined thermodynamic-stoichiometric models, in a semi-automated manner. This workflow includes the incorporation of physical and biochemical parameters of cellular compartments, automated definition of transporter variants and calculation of membrane transport Gibbs reaction energy, accurate addition of proton and charge balances, and addition of thermodynamic constraints for all reactions and transport processes. “Thermo-Flux” could automatically process 77% of the models in the BiGG database, ranging from bacteria to eukaryotic cells, and convert them into combined thermodynamic-stoichiometric models. While it failed for a minority of models for reasons mentioned above, we have demonstrated that limited additional manual effort can enable improved predictions of metabolic fluxes with models built by “Thermo-Flux”. This additional manual effort is essential to accurately represent more complex reactions and transport processes, because all reactions must be properly balanced for thermodynamic constraints to be reliable.

Currently, stoichiometric models rely on predefined reaction directions, which have been typically defined ad hoc, reflecting directions under canonical substrates, such as glucose. Relying on assumptions to define reaction directions tends to yield accurate flux predictions for such substrates, but these directions are not always guaranteed to be consistent with thermodynamic laws and experimental data, as demonstrated for the *E. coli* metabolic model. Additionally, such assumptions on reaction directions may not be transferable to other growth conditions, mutants or non-model organisms. On the other hand, thermodynamic constraints leverage first principles to define reaction directions and make stoichiometric models more accurate, which is particularly valuable when prior knowledge about reaction direction is limited. Using “Thermo-Flux”, thermodynamics constraints are integrated in a consistent manner, reducing the feasible solution space of combined thermodynamic-stoichiometric models and steering predictions toward biologically more meaningful states (Hädicke et al, 2018).

Several modeling considerations determine whether thermodynamic constraints can improve flux predictions in combined thermodynamic-stoichiometric models. First, these models are sensitive to physical and biochemical parameters such as pH and membrane potential differences, which influence the Gibbs reaction energy of transport processes and therefore dictate the direction in which protons and charge are transported across membranes. As stoichiometric network models often lack predefined directions for many transport processes (Zhang et al, 2024), thermodynamic constraints are particularly valuable for correctly constraining transporter directions and therefore obtaining valid flux predictions. Then, physical and biochemical parameters also determine the stoichiometry of protons and charge involved in each reaction, influencing the relative contribution of different metabolic pathways to the proton- and charge-balancing of the whole network (Shameer et al, 2018; Shaw and Cheung, 2019). Because incorrect pH- or charge-balancing can artificially generate energy that may act as an erroneous driving force for other reactions, organism-specific values for these parameters are essential for reliable flux predictions.

Second, thermodynamic-stoichiometric models also require definitions for upper and lower bounds on metabolite concentrations. As such, bounds together with the second law of thermodynamics define reaction directionality; a priori assumptions on reaction directions are not needed anymore in thermodynamic-stoichiometric models. Instead, users can define broad metabolite concentration ranges, e.g., from 0.1 μM to 10 mM, or narrower ranges based on metabolome measurements. The narrower the metabolite concentration ranges, the more constrained the flux space will be, which will translate into better flux predictions— provided that the measurements used are accurate, otherwise predictions will be incorrect as well. Advances in metabolomics now allow quantitative measurement of whole-cell concentrations of an increasing number of metabolites [preprint:(Canelas et al, 2011; Li et al, 2022; Wang et al, 2025)], and these measurements could be valuable to constrain reaction directions in a condition-specific manner.

Current limitations of using thermodynamic constraints relate to the assumptions made when estimating thermodynamic parameters. In fact, standard Gibbs formation energy estimates rely on the component contribution method (Noor et al, 2013) trained predominantly on aqueous-phase data at physiological temperatures, making estimates for untypical compounds (e.g., membrane-bound species) or conditions (e.g., extreme temperatures) less reliable. To improve thermodynamic parameter estimates, recent group-contribution and molecular thermodynamic models could be incorporated into the estimation workflow, for instance, to more accurately capture the temperature dependence of standard Gibbs formation energies (Du et al, 2018) or to better account for the non-aqueous conditions of membrane compartments (Panayiotou et al, 2016). Expanding experimental datasets covering a broader range of compounds and temperature would further strengthen the reliability of thermodynamic parameter estimates. Together, these measures would extend the applicability of thermodynamic constraints to non-aqueous compartments and membrane-bound metabolites, or less common organisms such as extremophiles.

Looking ahead, we anticipate that models built with “Thermo-Flux” will advance both fundamental and applied research. On the fundamental side, models with a stronger mechanistic foundation are required for better predictions. Beyond improved mechanistic accuracy, models augmented with “Thermo-Flux” can address several challenges: First, Gibbs energies estimated by the component contribution method still contain uncertainties. Through fitting thermodynamic-stoichiometric models to extracellular fluxes and metabolome data, it should be possible to further narrow down such uncertainties and to adjust standard Gibbs energies to in vivo conditions. Second, by explicitly modeling dependence on physical and biochemical parameters (pH, ionic strength, temperature, and membrane potential) models augmented with “Thermo-Flux” link flux predictions to cellular conditions and thus allow systematic sensitivity analyses to physical and biochemical parameters. Such analyses pave the way to investigating energetic and mechanistic features of proton- and charge-coupled pathways (Shameer et al, 2018; Shaw and Cheung, 2019). Third, models developed with “Thermo-Flux” facilitate the analysis of thermodynamic principles on the genome-scale, from candidate cellular objectives to relationships such as the trade-off between yield and growth rate (Lipson, 2015; Saadat et al, 2020), upper bounds on Gibbs-energy dissipation (Niebel et al, 2019), the flux-force relationship (Beard and Qian, 2007), and the max-min driving force (MDF) (Hädicke et al, 2018; Noor et al, 2014). On the applied side, thermodynamic-stoichiometric models developed with “Thermo-Flux” can support biotechnology and metabolic engineering through improved predictive capabilities and the possibility of uncovering thermodynamic bottlenecks.

## Methods

### Implementing conditional constraints in a linear program

The second law constraint relies on a conditional statement over the sign of the flux *v*. In linear programming, such constraints are implemented by introducing an auxiliary binary variable *b* as the indicator of the sign.

$$\left\{\begin{array}{l}v\, > \, 0\Rightarrow b=1\quad \hfill \\ v\le 0\Rightarrow b=0\quad \end{array}\right.$$

Therefore, the sign of the flux *v* depends on the value of the binary variable, which is enforced by implementing the following constraints.

$$\left\{\begin{array}{c}v\ge M\cdot (b-1)\\ v\le M\cdot b\hfill \end{array}\right.\,$$

The value of M should be chosen as tight as possible to avoid numerical issues (Gurobi Optimization, LLC, 2023). By default, in “Thermo-Flux”, it is equal to the largest magnitude of the bounds on v.

To model the second law, we apply the same logic on the *Δ*_*r*_*G* variable.

$$\left\{\begin{array}{l}{\Delta }_{r}G > 0\Rightarrow b=0\quad \hfill \\ {\Delta }_{r}G\le 0\Rightarrow b=1\quad \end{array}\right.$$

As done previously, this conditional constraint is enforced by:

$$\left\{\begin{array}{c}{\Delta }_{r}G\le {M}^{{\prime} }\cdot b\hfill \\ {\Delta }_{r}G\ge {M}^{{\prime} }\cdot (b-1)\end{array}\right.\,$$

where M’ is this time larger than the maximum possible magnitude of *Δ*_*r*_*G* to avoid numerical issues.

### Gibbs energy dissipation rate

The Gibbs energy dissipation rate is the sum of all exchange reactions’ Gibbs reaction energy multiplied by the corresponding reaction flux. It is implemented by defining a new variable *g*_diss_ that equals this sum (Niebel et al, 2019).

$${g}_{diss}={\sum}_{j}{\Delta }_{r}{G}_{j}\cdot {v}_{j}$$

where j is an exchange reaction.

An upper bound can be applied to *g*_diss_:

$${g}_{diss}\le \,{g}_{\lim }\quad \,{{\mbox{in}}}\,\quad J\cdot gD{W}^{-1}\cdot {h}^{-1}$$

### Balancing three-compartment transport reactions

“Thermo-Flux” balancing algorithm was developed to handle metabolic reactions and transport processes which occur between two compartments. To extend the use of the balancing algorithm to handle three-compartment reactions, such as those mediated by the TON motor complex in Gram-negative bacteria (Ratliff et al, 2022), we adopted the following approach. When a three-compartment reaction is provided, the balancing algorithm decomposes it into a set of sub-transport reactions and a core reaction. Sub-transport reactions are identified by detecting metabolites that move between compartments without chemical transformation—in practice, this detects metabolites whose identifier is unchanged when the compartment annotation is removed. These metabolites are extracted from the original reaction, yielding a reduced core reaction. The two-compartment balancing algorithm is then applied independently to each sub-transport reaction and to the core reaction.

For this decomposition to be valid, the core reaction must span no more than two compartments after removing the transported metabolites. This means that this approach is not applicable in two cases: (i) reactions in which a metabolite is chemically transformed during transport, since the transported metabolites cannot be clearly distinguished and separated, and (ii) reactions spanning more than three compartments. The handling of these cases is currently not supported and remains future work.

#### Applying “Thermo-Flux” on models from the BiGG database

We downloaded all models stored in the BiGG database (version “1.6.0”) via the BiGG Models web API. We downloaded the models in the “.xml” format. We used “Thermo-Flux” to convert every model into a combined thermodynamic-stoichiometric model. We set biomass production as the objective function and erased all prior reaction directions.

Growth rate predictions with FBA (stoichiometric constraints only) were obtained using COBRApy functions. Then, thermodynamic constraints were incorporated using tmodel.add_TFBA_variables(), and the growth rate was predicted again. Predicted growth rates below 10^−6^ were attributed to numerical errors and considered as being zero. All FBA calculations with thermodynamic constraints were performed on the university’s computational cluster *Hábrók*, with each optimization allowed to run until an optimal solution was found or a maximum wall time of 24 h was reached.

#### *E. coli* model conversion and analysis

The stoichiometric model used here is based on the iJR904 reconstruction (Niebel et al, 2019), with modifications to several transporter variants relative to the BiGG reconstruction; a complete list of changes is provided elsewhere (Niebel et al, 2019). We used the values of physical and biochemical parameters as defined in (Niebel et al, 2019): a pH of 7.0 in the extracellular space, and 7.6 in the cytosol, an ionic strength of 0.15 M for the cytosol, and 0.2 M for the extracellular medium and a transmembrane potential of 150 mV across the cell membrane (cf. Appendix Table S2). Blocked reactions (i.e., reactions that cannot carry any flux under the stoichiometric constraints) and orphaned metabolites (i.e., metabolites that do not participate in any reaction that can carry flux) were identified and removed using the built-in COBRApy function.

Using metabolome data (Kochanowski et al, 2017), we formulated bounds on metabolite concentration for 34 metabolites. Bounds were formulated such that the concentrations remain within the 99% confidence interval of the measured values (e.g., *c*_*max*_ = *μ* + 2.96*σ with *c*_max_ being the upper bound on concentration, *μ* the mean measured value and *σ* the reported standard deviation). Similarly, measured physiological data (Vila Chã Losa, 2024) was used to apply bounds on exchange fluxes that were non-zero in the experimental data, using a 95% confidence interval.

Instead of setting upper and lower bounds to measured CO_2_ excretion and O_2_ uptake rates, we constrained the model using an upper and lower bound on the respiratory quotient. We defined the upper and lower bound by using the mean measured respiratory quotient ${\mu }_{RQ}=\frac{{v}_{C{O}_{2}}}{{v}_{{O}_{2}}}$ and calculated its uncertainty with the following formula:

$${\sigma }_{RQ}=RQ\cdot \sqrt{{\left(\frac{{\sigma }_{{O}_{2}}}{{v}_{{O}_{2}}}\right)}^{2}+{\left(\frac{{\sigma }_{C{O}_{2}}}{{v}_{C{O}_{2}}}\right)}^{2}}$$

where ${v}_{{O}_{2}}$ and ${\sigma }_{{O}_{2}}$ are the mean and uncertainty of the O_2_ exchange flux and ${v}_{C{O}_{2}}$ and ${\sigma }_{C{O}_{2}}$ for the *CO*_2_ exchange flux. The bounds on the respiratory quotient were set to *μ*_RQ_ ± 6 ⋅ *σ*_RQ_.

We performed variability analyses with the function implemented in “Thermo-Flux”: solver.gurobi.variability_analysis(). Each reaction was classified as forward, backward, reversible, or blocked based on its range of feasible fluxes under thermodynamic constraints.

#### Predictions of exchange fluxes for low-glucose conditions in *E. coli*

Exchange fluxes under low-glucose conditions were predicted for *E. coli* using two approaches: standard FBA with a stoichiometric model (iJR904) with predefined reaction directions, and FBA with thermodynamic constraints and measured metabolome data to constrain metabolite concentrations. In both cases, biomass production was set as the objective function, and the glucose uptake rate of the model was set to the experimentally measured one.

#### Predictions of exchange fluxes using FBA with thermodynamic constraints in *S. cerevisiae*

To predict exchange fluxes across a broad range of physiological conditions in *S. cerevisiae*, including conditions exhibiting overflow metabolism, we used FBA with thermodynamic constraints and the converted iMM904 model. We set biomass production as the objective function, and we constrained the cellular Gibbs energy dissipation rate with an upper limit *g*_*lim*_ = 3700*J* ⋅ gDW^−1^ ⋅ h^−1^ as taken from (Niebel et al, 2019). Then, we performed FBA with thermodynamic constraints over the different physiological conditions by setting the glucose uptake rate to the experimentally measured one.

## Supplementary information

Appendix

Table EV1

Table EV2

Table EV3

Peer Review File

Expanded View Figures

## Data Availability

The package produced in this study is available on GitHub: https://github.com/molecular-systems-biology/thermo-flux. The source data of this paper are collected in the following database record: biostudies:S-SCDT-10_1038-S44320-026-00227-4.
