# Supplementary material for: Thermo-flux: generation and analysis of thermodynamic-stoichiometric metabolic network models
Source: Mol Syst Biol. 2026 Jul 3;22(10):1650–64. doi: 10.1038/s44320-026-00227-4 (PMC13639002; doi:10.1038/s44320-026-00227-4)
Supplement: Supplementary file 1 [file 44320_2026_227_MOESM1_ESM.pdf]

Appendix for Thermo-Flux: generation and analysis of thermodynamic-stoichiometric metabolic network models

Table of Contents

Appendix Table S1	Code snippets : illustration of the protocol steps for the conversion of the iMM904 model	1
Appendix Table S2	Code snippets : specific correction steps for the curation of the *E. coli* model	2

Appendix Table S1 Code snippets : illustration of the protocol steps for the conversion of the iMM904 model

Appendix Table S1: Detail of the steps required to convert the iMM904 model into a combined thermodynamic-stoichiometric model. The code snippets illustrate the steps mentioned in the manuscript.

n°	Code	Explanation
1.1	`model.pH = {'c': Q_(7), 'e': Q_(5),`<br>`'v': Q_(5.5), 'm': Q_(7.4)}`<br>`model.I = {'c': Q_(0.25, 'M'),'e': Q_(0.25, M')}`<br>`model.phi = {'ce': Q_(0.06, 'V'), 'cm': Q_(0.16, 'V')}`<br>`model.T = Q_(298, 'Kelvin')`	Setting physical and biochemical parameters.
2.1	`metabolite.average_charge_protons()`	Query the average charge and proton of a reactant.
2.2	`thermo_flux.tools.drg_tools.pka_graph(metabolite,`<br>`pMg , ionic_strength , temperature).plot()`	Inspect the resulting species distribution of a reactant.
2.3	`tmodel.metabolites.focytc_m.charge = 2`<br>`tmodel.metabolites.ficytc_m.charge = 3`	Update the charge of the ferro-cytochrome redox couple.
3.1	`tmodel.update_thermo_info()`	Calculate reaction Gibbs energies for all metabolites of the iMM904 model.
4.1	`thermo_flux.tools.drg_tools.add_transporter_variants`<br>`(tmodel.reactions.PIt2m,`<br>`balance_charge=True, add_charge_neutral=True)}`	Add a charge neutral variant for all phosphate transporters.
4.2	`tmodel.reactions.ATPS.transported_h =`<br>`{'c': -1.0, 'e' : 1}`	Specify the number of transported protons in the cytosolic ATPase (ATPS).
4.3	`tmodel.reactions.DOLPMTcer.transported_mets =`<br>`{tmodel.metabolites.dolmanp_r: -1}`	Specify the transported metabolite in DOLPMTcer.
5.1	`thermo_flux.tools.drg_tools.reaction_balance(`<br>`reaction, balance_charge=True)`	Balance all reactions in the iMM904 model.
7.1	`tmodel.add_TFBA_variables()`	Setup a thermodynamically constrained FBA optimization problem of the iMM904 model.
7.2	`tmodel.m.optimize()`	Optimize the model using the Gurobi solver.
B2	`tmodel.reactions.rxn_id.ignore_snd = True`	Ignore the second law constraint for the given reaction. The reversibility of this reaction is thus only determined by its flux bounds. "rxn_id" must be replaced by the id of the reaction.
B3	`tmodel.regression()`	Add regression constraints and objectives to the previously constructed FBA problem with thermodynamic constraints.

Appendix Table S2 Code snippets : specific correction steps for the curation of the *E. coli* model

Appendix Table S2: Curation steps applied to the *E. coli* model. Manual intervention was required for several transport reactions in which the transported metabolite could not be automatically identified by *Thermo-Flux*, as well as for reactions whose stoichiometry was found to be incorrect and needed to be updated.

Reaction	Code	Explanation
Formate dehydrogenase	`FDH2.add_metabolites({h_e:2, h_c:-2, h2o_c:1, h2o_e:-1})` `FDH3.add_metabolites({h_e:2, h_c:-2, h2o_c:1, h2o_e:-1})`	These two formate dehydrogenases were incorrectly defined in the original iJR904 model. We added proton translocation and corrected the compartments of the water metabolites.
Dimethyl sulfoxide reductase	`DMSOR1e.add_metabolites({h_e:-2, h_c:2})` `DMSOR1e.transported_mets = {dmso_e: -1}` `DMSOR2e.add_metabolites({h_e:-2, h_c:2})` `DMSOR2e.transported_mets = {dmso_e: -1}`	Define DMSO as transported species and correct proton stoichiometry.
Trimethylamine N-oxide reductase	`TMAOR1e.transported_mets = {tmao_e: -1}` `TMAOR2e.transported_mets = {tmao_e: -1}`	Define TMAO as transported species.
Glucose dehydrogenase	`GLCDe.transported_mets = {glc-D_e: -1}`	Define glucose as transported species.
Sirohydrochlorin ferrochelatase	`SHCHF.add_metabolites({scl_c:-1, srch_c:1})` `srch_c.annotation = {'bigg.metabolite': 'dscl'}`	Correct stoichiometric coefficients and update annotations.
Sirohydrochlorin dehydrogenase	`SHCHD2.add_metabolites({scl_c:2, srch_c:-2})`	Correct stoichiometric coefficients.
NMN transport via NMN glycohydrolase	`NMNt7.transported_mets = {nmn_e: -1}`	Define chemical transformation of NMN as part of the transport mechanism.

Note: For readability, the prefix `model.reactions.` is omitted in all code snippets of the *E. coli* model curation above.
