# Supplementary material for: Thermo-flux: generation and analysis of thermodynamic-stoichiometric metabolic network models
Source: Mol Syst Biol. 2026 Jul 3;22(10):1650–64. doi: 10.1038/s44320-026-00227-4 (PMC13639002; doi:10.1038/s44320-026-00227-4)
Supplement: Supplementary file 2 [file 44320_2026_227_MOESM5_ESM.pdf]

Thermo-Flux: generation and analysis of thermodynamic-stoichiometric metabolic network models

Edward Smith, Nathan Fargier, José Pedro, and Matthias Heinemann

Corresponding author(s): Matthias Heinemann (m.heinemann@rug.nl)

Review Timeline:

Submission Date:	25th Nov 25
Editorial Decision:	9th Jan 26
Revision Received:	29th Apr 26
Editorial Decision:	1st Jun 26
Revision Received:	3rd Jun 26
Accepted:	10th Jun 26

Editor: Jingyi Hou

Transaction Report:

(Note: With the exception of the correction of typographical or spelling errors that could be a source of ambiguity, letters and reports are not edited. Depending on transfer agreements, referee reports obtained elsewhere may or may not be included in this compilation. Referee reports are anonymous unless the Referee chooses to sign their reports.)

9th Jan 2026

Manuscript Number: MSB-2025-13491
Title: Thermo-Flux: generation and analysis of comprehensive thermodynamic-stoichiometric metabolic network models
Author: Edward Smith
Nathan Fargier
José Pedro
Matthias Heinemann

Dear Prof Heinemann,

Thank you for submitting your work to Molecular Systems Biology. First, I would like to apologize for the delay in the review process, which was due to the holiday season. We have now received evaluations from the three reviewers who agreed to assess your manuscript.

As you will see from the comments below, both Reviewer #1 and Reviewer #2 recognize the potential and interest of the presented method, while raising questions regarding methodological details and validation. Reviewer #3, on the other hand, expressed concerns about the method itself and its advancement over existing approaches.

Given the overall balance of these assessments, we invite you to submit a revision of your manuscript. As you may already know, our editorial policy allows in principle a single round of major revision so it is essential to provide responses to the reviewers' comments that are as complete as possible. Please feel free to contact me in case you would like to discuss in further detail any of the issues raised by the reviewers.

On a more editorial level, we would ask you to address the following issues:

- Please provide a .docx formatted version of the manuscript text (including legends for main figures, EV figures and tables). Please make sure that the changes are highlighted to be clearly visible.

- Please provide individual production quality figure files as .eps, .tif, .jpg (one file per figure). When assembling figures, please refer to our figure preparation guideline in order to ensure proper formatting and readability in print as well as on screen: https://media.springernature.com/original/springer-cms/rest/v1/content/27825798/data/v1

-Please provide a .docx formatted letter INCLUDING the reviewers' reports and your detailed point-by-point responses to their comments. As part of the EMBO Press transparent editorial process, the point-by-point response is part of the Review Process File (RPF), which will be published alongside your paper.

-Please note that all corresponding authors are required to supply an ORCID ID for their name upon submission of a revised manuscript.

-We replaced Supplementary Information with Expanded View (EV) Figures and Tables that are collapsible/expandable online. A maximum of 5 EV Figures can be typeset. EV Figures should be cited as 'Figure EV1, Figure EV2" etc... in the text and their respective legends should be included in the main text after the legends of regular figures.

Additional Tables/Datasets should be labeled and referred to as Table EV1, Dataset EV1, etc. Legends have to be provided in a separate tab in case of .xls files. Alternatively, the legend can be supplied as a separate text file (README) and zipped together with the Table/Dataset file.

For the figures and tables that you do NOT wish to display as Expanded View figures, they should be bundled together with their legends in a single PDF file called *Appendix*, which should start with a short Table of Content. Each legend should be below the corresponding Figure/Table in the Appendix. Appendix figures and tables should be referred to in the main text as: "Appendix Figure S1, Appendix Figure S2, Appendix Table S1" etc. See detailed instructions regarding expanded view here: https://link.springer.com/journal/44320/submission-guidelines#cms-Expanded-View-data.

-Before submitting your revision, primary datasets (and computer code, where appropriate) produced in this study need to be deposited in an appropriate public database.
Please remember to provide a reviewer password if the datasets are not yet public.
The accession numbers and database should be listed in a formal "Data Availability" section (placed after Materials & Method) that follows the model below (see also https://link.springer.com/partners/embo-press/editorial-policies#Data%20availability%20statement). Please note that the Data Availability Section is restricted to new primary data that are part of this study.

Data availability
The datasets (and computer code) produced in this study are available in the following databases:
- RNA-Seq data: Gene Expression Omnibus GSE46843 (https://www.ncbi.nlm.nih.gov/geo/query/acc.cgi?acc=GSE46843)
- [data type]: [name of the resource] [accession number/identifier/doi] ([URL or identifiers.org/DATABASE:ACCESSION])
*** Note - All links should resolve to a page where the data can be accessed. ***

- At EMBO Press we ask authors to provide source data for the main manuscript figures. You will receive a separate email with instructions for providing source data with your revised manuscript, including how to upload and organize the files.
Additional information on source data and instruction on how to label the files are available < https://link.springer.com/journal/44320/submission-guidelines#cms-Source-data >

- Our journal encourages inclusion of *data citations in the reference list* to directly cite datasets that were re-used and obtained from public databases. Data citations in the article text are distinct from normal bibliographical citations and should directly link to the database records from which the data can be accessed. In the main text, data citations are formatted as follows: "Data ref: Smith et al, 2001". In the Reference list, data citations must be labeled with "[DATASET]". A data reference must provide the database name, accession number/identifiers and a resolvable link to the landing page from which the data can be accessed at the end of the reference. Further instructions are available at < https://link.springer.com/journal/44320/submission-guidelines#cms-Reference-guidelines >.

- We updated our journal's competing interests policy in January 2022 and request authors to consider both actual and perceived competing interests. Please review the policy (https://link.springer.com/partners/embo-press/editorial-policies#Competing%20interest%20disclosures) and update your competing interests if necessary.
Please use the heading "Disclosure statement and competing interests".

- All Materials and Methods need to be described in the main text using our 'Structured Methods' format. According to this format, the Methods section includes a Reagents and Tools Table (listing key reagents, experimental models, software and relevant equipment and including their sources and relevant identifiers) followed by a Methods and Protocols section describing the methods, ideally using a step-by-step protocol format. The aim is to facilitate adoption of the methodologies across labs.

Please download and fill our Reagents and Tools Table template (.docx), which you can find in our author guidelines: https://link.springer.com/journal/44320/submission-guidelines#structuredmethods.

When submitting your revised manuscript, please do not include the Reagents and Tools Table in the Methods section of the manuscript but upload it as a separate file choosing the file type "Reagent Table".

-Regarding data quantification:
Please ensure to specify the name of the statistical test used to generate error bars and P values, the number (n) of independent experiments (please specify technical or biological replicates) underlying each data point and the test used to calculate p-values in each figure legend. Discussion of statistical methodology can be reported in the materials and methods section, but figure legends should contain a basic description of n, P and the test applied.
Graphs must include a description of the bars and the error bars (s.d., s.e.m.).
Please also include scale bars in all microscopy images.

- Please provide a "standfirst text" summarizing the study in one or two sentences (approximately 250 characters, including space), three to four "bullet points" highlighting the main findings and a " visual abstract" (550px width and 400-600 px height, PNG format) to highlight the paper on our homepage.
Please refer to published papers for examples.

When you resubmit your manuscript, please download our CHECKLIST (https://media.springernature.com/original/springer-cms/rest/v1/content/27825796/data/v1) and include the completed form in your submission.
Please note that the Author Checklist will be published alongside the paper as part of the transparent process .

If you feel you can satisfactorily deal with these points and those listed by the referees, you may wish to submit a revised version of your manuscript. Please attach a covering letter giving details of the way in which you have handled each of the points raised by the referees. A revised manuscript will be once again subject to review and you probably understand that we can give you no guarantee at this stage that the eventual outcome will be favorable.

I look forward to receiving the revised manuscript soon.

Kind regards,
Jingyi

Jingyi Hou, PhD

Senior Editor
Molecular Systems Biology

We realize that it is difficult to revise to a specific deadline. In the interest of protecting the conceptual advance provided by the work, we recommend a revision within 3 months (9th Apr 2026). Please discuss the revision progress ahead of this time with the editor if you require more time to complete the revisions. Use the link below to submit your revision:

https://msb.msubmit.net/cgi-bin/main.plex

IMPORTANT: Please read our guidelines for revised submissions: https://link.springer.com/journal/44320/submission-guidelines#cms-Revised-submissions

*** PLEASE NOTE *** As part of the EMBO Press transparent editorial process initiative (see our Editorial at https://dx.doi.org/10.1038/msb.2010.72), Molecular Systems Biology publishes online a Review Process File with each accepted manuscripts. This file will be published in conjunction with your paper and will include the anonymous referee reports, your point-by-point response and all pertinent correspondence relating to the manuscript. If you do NOT want this File to be published, please inform the editorial office at contact@molsystbiol.org within 14 days upon receipt of the present letter.

Reviewer #1:

In this manuscript, Heinemann and colleagues introduce Thermo-Flux, a computational package that integrates thermodynamic constraints into stoichiometric metabolic network models. This semi-automated tool improves predictions of metabolic fluxes by incorporating Gibbs formation energies, protonation states, transport processes, and biomass thermodynamics into genome-scale metabolic models. The authors apply Thermo-Flux to 87 models from the BiGG database, demonstrating its ability to refine flux predictions and reveal thermodynamically constrained solutions. The manuscript provides a well-documented methodology that promises to enhance the predictive power of metabolic models, making it a valuable tool for systems biology and metabolic engineering. The manuscript is generally well-written.

Major

The manuscript would benefit from a more detailed presentation of how incorporating thermodynamic constraints influences model predictions. Providing this would better demonstrate the practical utility and novelty of the method compared to existing approaches like OptMDFpathway (PMID: 30248096 PMCID: PMC6171959 DOI: 10.1371/journal.pcbi.1006492) and ETGEMs (PMID: 34174426 DOI: 10.1016/j.ymben.2021.06.005), which also integrate thermodynamics but do not account for transport processes or pH differences across cellular compartments. Additionally, these methods provide valuable insights into pathway and target predictions. Currently, the analysis primarily focuses on solution space compression and growth fitting, but a comparison with the baseline model would help to more clearly highlight how the optimization of parameter acquisition contributes to improved model performance.

The automation of thermodynamic constraint application is a notable contribution, but it would be helpful for the authors to discuss why it is less automated than established protein resource constraint models. Given that thermodynamic constraints typically have lower data variability, the automation of this step could potentially be more robust and may benefit from further refinement.

It is unclear whether the Gibbs formation energies generated in Thermo-Flux are based on the same computational methods as those used in eQuilibrator. It would be beneficial for the authors to clarify this point and explain any differences between calculating metabolite formation energies with Thermo-Flux versus using eQuilibrator to generate parameters.

Biomass formation is treated as a metabolite with an associated Gibbs formation energy in the model. However, the authors should note that biomass formation is not a simultaneous process but rather a sequential one, meaning it does not strictly adhere to steady-state assumptions. Assigning a Gibbs formation energy to biomass formation may not be appropriate, and the synthesis of biomass could be excluded from thermodynamic constraints, as it does not fully align with the concept of simultaneous reactions and steady-state flux balancing.

Minor

Figure 1 could benefit from a clearer organization and a more logical flow of steps. A more intuitive layout would help readers better follow the described workflow.

The manuscript does not address the temperature dependency of the thermodynamic parameters. Since temperature can influence Gibbs energies, it would be useful to mention this limitation, particularly in the context of heat-tolerant organisms or industrial applications that may operate under extreme conditions.

Reviewer #2:

The authors present Thermo-flux, a software tool that automates the construction of Gibbs free energy-based thermodynamic constraints for genome-scale metabolic models (GEMs) and enables flux balance analysis (FBA) under those constraints. Thermodynamic constraints are known to reduce the feasible solution space and are expected to steer predictions toward biologically more meaningful states. However, assembling consistent free-energy bounds and implementing thermodynamics-constrained FBA (FBA,ST) is non-trivial. By addressing both tasks in a single workflow, Thermo-flux has the potential to broaden access to FBA,ST for routine users. In addition, FBA, ST automatically integrates metabolomic data with GEMs when such data is available. The study is timely and the software could be impactful. At the same time, in its current form it is difficult for me to assess the tool's practical usefulness and robustness. The manuscript would benefit from clearer methodological detail and stronger validation. Below are comments and suggestions to improve the paper.

MAJOR COMMENTS

1) According to the Methods, it takes Thermo-flux FBA,S,T up to 24 hours to complete a single run with an average model:

"The calculations for (FBA - S,T) were run on the university's computational cluster 'Hábrók' and were optimized until the optimal solution was found, with a maximal duration of 24 h each."

If I understand correctly, this refers only to the mixed-integer linear optimization after all constraints are set. By comparison, parsimonious flux balance analysis (pFBA), which extends FBA by adding a secondary objective to minimize the total flux and thereby removes futile cycles, typically solves in seconds on standard GEMs. While Thermo-flux offers more than just eliminating loops, a 24-hour solve time is a serious disadvantage relative to routine approaches. An alternative semi-standard method is loopless FBA.

My questions related to this issue are:
(1a) Is it true that FBA,S,T takes about 24 hours for the optimization step alone? Please clarify whether this is a typical runtime or a maximal wall-clock limit, and under what settings.
(1b) How long does Flux Variability Analysis (FVA) take under S,T constraints? Since FVA requires solving many optimizations (one or two per reaction), a full analysis with FBA,S,T and FVA,S,T could be impractically long; please provide typical runtimes.
(1c) How does the optimization time compare with previously established loopless FBA algorithms on the same models and hardware?

2) The validations presented (Figure 6) are underwhelming, and it is not clear from these results whether Thermo-flux optimizations are superior or useful, or in what specific ways.
2a) The decrease in flux ranges from routine FBA to FBA,S,T in Fig. 6b appears minimal overall. Specific examples would be useful to demonstrate how FBA, ST is useful.
2b) The numbers in Fig. 6c report only how many changes occurred. Again, specific examples showing that the results change in a positive way would be useful.
2c) Fig. 6c, showing very accurate predictions of exchange fluxes, could be a strong validation, but it needs benchmarking against standard approaches: classical FBA, pFBA, and loopless FBA.

3) An important feature of Thermo-flux is the straightforward integration of metabolite concentrations when metabolomics data are available. This is not demonstrated by an example. It does not need to be network-scale. A small demonstration showing how narrowing metabolite concentration bounds affects ΔG′{degree sign}, reaction directionality, and flux ranges would be useful to illustrate the practical impact.

MINOR COMMENTS (in the order of importance)

1) Page 8: addition of the artificial metabolite "charge" for charge balancing of compartments.

It is not clear what this achieves. Transport reactions that only exchange ions like Na+ can balance out the "charge" metabolite in a straightforward manner with no mathematical effect on the solution. Please correct me if I am wrong and clarify the usefulness of this approach.

2) Page 10: Equation 9.

From the equation and its brief explanation, it appears to multiply precomputed constant free-energy values by fluxes, which

would be linear (meaning the optimization problem would remain convex). I think the problem becomes non-convex due to mixed-integer variables as explained in the methods, but this should be clearly stated in the main text.

3) Page 10:"An upper limit in the cellular Gibbs energy dissipation rate"
It is never mentioned what the value of this limit is.

4) Page 14: "While it failed for a minority of models for reasons mentioned above, we have demonstrated with the iMM904 model that with limited additional manual effort, 'Thermo-Flux' can convert large stoichiometric models into combined thermodynamic stoichiometric models that enable improved predictions of metabolic fluxes."

It is not clear what is meant by additional manual effort here.

5) Page 5, first sentence: "where NH is the number of hydrogen atoms in the species"

I think what the authors mean is the number of hydrogen ions that can be gained or lost, not the total H atoms in a compound.

6) Page 3: "pMg = 3.0,"

This is not consistent with the number in Table 1, which indicates 14

7) Box 1: Please explain when photon energy can be useful in metabolic modeling.

Reviewer #3:

The authors present Thermo-Flux, an automated pipeline to reconstruct thermodynamically constrained stoichiometric models. The pipeline is designed to constrain reaction directionalities in stoichiometric models (e.g., genome-scale metabolic models) according to the second law of thermodynamics. While similar tools exist for this purpose, this pipeline in particular offers automated charge/proton balancing and the calculation of thermodynamic properties for transport reactions and uncommon reactions such as growth. The reviewer agrees that presenting a fully automated pipeline, compatible with recent advances in machine learning and data science to account for thermodynamics in metabolic models, is significant and potentially impactful. However, the current pipeline proposed by the authors has important shortcomings and does not offer a substantial improvement over previously published methods.

Major comments

1. One of the main contributions of this paper is the proposal of an automated pipeline for charge/proton balancing of reactions. However, the method presented by the authors is confusing and results in reactions with non-integer stoichiometric coefficients. Even if one assumes that users do not need to fully understand the underlying balancing mechanism and only need to apply the tool, they must ultimately be able to interpret the resulting reactions. When users inspect the final model, they should have a clear sense of what these reactions represent, which is not the case with the current formulation.

2. Along the same lines, I propose the following alternative approach to reaction balancing. Starting from the example presented in the paper, let us assume a reaction involving phosphate as one of the reactants, and that phosphate exists in two forms at the given pH, namely PO_4^{3-} and HPO_4^{2-}. Instead of the formulation presented by the authors, the original reaction could be rewritten as the following set of reactions:

a. $PO_4^{3-} + A \rightarrow B$

b. $HPO_4^{2-} + A \rightarrow B'$

c. $H^+ + PO_4^{3-} \leftrightarrow HPO_4^{2-}$

Assuming a constant steady-state pH, all reactions are mass- and charge-balanced. Moreover, if one wishes to enforce a specific ratio between the two phosphate species, similar to what the authors aim to achieve, this can be done through constraints on metabolite concentrations. This formulation is easier to interpret and does not require the additional assumptions and complexity introduced by the authors' approach.

3. The authors mention differences in metabolite concentrations across cellular compartments. This is an important consideration, but it was not clear whether or how this was incorporated into their formulation. The authors should clarify this point.

4. Page 12, second paragraph of the Results section, should be improved. Rather than stating the obvious, the authors should analyze these results in more depth. Which reactions were over-constrained? What were the underlying reasons (e.g., errors in Gibbs energy estimation, incorrect balancing, uncertainty in reaction participants)? Are there insights from these cases that could be used to improve the proposed pipeline?

Minor comments

1. In Box 2, the authors state that for paired metabolites lacking an explicit biosynthesis pathway (e.g., NAD(P)/NAD(P)H), they set the concentration of one cofactor to 1 M. What do the authors mean by "explicit biosynthesis pathway"? Organisms such as E. coli or yeast can synthesize these compounds from simple carbon sources like glucose. Moreover, if these compounds participate in other reactions, setting their concentration to such a high value could introduce inconsistencies or artifacts in the overall simulations.

2. In my experience, the impact of integrating thermodynamic constraints is particularly evident for reactions involving novel

compounds (e.g., xenobiotics), as the directionality of native and common reactions is usually well curated. Can this pipeline be applied to reactions involving novel compounds, especially those not present in the eQuilibrator database? The authors should comment on this point and discuss the potential requirements or limitations of their approach in such cases.

3. In some eukaryotic cells, for example yeast-which the authors use as a demonstration case-metabolism occurs in multiple membranous compartments. Most equations used to calculate thermodynamic properties are developed for aqueous conditions, whereas membrane environments do not readily satisfy these assumptions. Did the authors make any special considerations for reactions occurring in or across membranes?

Thermo-Flux response to reviewer

Editor's comment:

As you will see from the comments below, both Reviewer #1 and Reviewer #2 recognize the potential and interest of the presented method, while raising questions regarding methodological details and validation. Reviewer #3, on the other hand, expressed concerns about the method itself and its advancement over existing approaches.

Given the overall balance of these assessments, we invite you to submit a revision of your manuscript. As you may already know, our editorial policy allows in principle a single round of major revision so it is essential to provide responses to the reviewers' comments that are as complete as possible. Please feel free to contact me in case you would like to discuss in further detail any of the issues raised by the reviewers.

Reviewer #1:

In this manuscript, Heinemann and colleagues introduce Thermo-Flux, a computational package that integrates thermodynamic constraints into stoichiometric metabolic network models. This semi-automated tool improves predictions of metabolic fluxes by incorporating Gibbs formation energies, protonation states, transport processes, and biomass thermodynamics into genome-scale metabolic models. The authors apply Thermo-Flux to 87 models from the BiGG database, demonstrating its ability to refine flux predictions and reveal thermodynamically constrained solutions. The manuscript provides a well-documented methodology that promises to enhance the predictive power of metabolic models, making it a valuable tool for systems biology and metabolic engineering. The manuscript is generally well-written.

Major

The manuscript would benefit from a more detailed presentation of how incorporating thermodynamic constraints influences model predictions. Providing this would better demonstrate the practical utility and novelty of the method compared to existing approaches like OptMDFpathway (PMID: 30248096 PMCID: PMC6171959 DOI: 10.1371/journal.pcbi.1006492) and ETGEMs (PMID: 34174426 DOI: 10.1016/j.ymben.2021.06.005), which also integrate thermodynamics but do not account for transport processes or pH differences across cellular compartments. Additionally, these methods provide valuable insights into pathway and target predictions. Currently, the analysis primarily focuses on solution space compression and growth fitting, but a

comparison with the baseline model would help to more clearly highlight how the optimization of parameter acquisition contributes to improved model performance.

We understand the reviewer's point. In general, "Thermo-Flux" generates mechanistically more accurate models, with (i) explicitly balancing chemical species, protons, and charges in a pH-dependent manner, (ii) detailed modeling of the thermodynamics of transport processes, and (iii) definition of reaction reversibility by means of the second law. In addition, the models generated with "Thermo-Flux" can also be constrained by metabolome data.

We see the following improvements that a thermodynamic-stoichiometric model generated with Thermo-Flux can achieve compared to what now exists in the field:

1) Stoichiometric models have predefined directions for certain metabolic reactions. These reaction directions have typically been defined using prior « knowledge ». Often, these *a priori* defined reaction directions correspond to the ones that would occur on canonical growth substrates, such as glucose. However, it does not mean that these *a priori* defined reactions directions are correct (as we now show in this manuscript), nor that they hold across other growth conditions. With thermodynamic-stoichiometric models generated by Thermo-Flux, reaction directions are constrained by the second law of thermodynamic, based on first principles and in a condition-dependent manner, which leads to mechanistically more correct models.
2) Even if current stoichiometric models have been augmented to include the 2nd law of thermodynamics, they do not consider proper pH-balancing, chemical species and accurate modeling of the thermodynamics of transport processes. Thermo-Flux implements these aspects in a semi-automatic manner, further improving the mechanistic accuracy of constrained-based models.

We argue that mechanistically more correct models are at the very basis of *better* predictions, even though there might be growth conditions where these additional constraints are not "active". The question on whether a mechanistically more correct model leads to better predictions also depends on the baseline model that one uses for comparison. For example, if a model was manually curated to be properly balanced under typical biochemical and physical conditions (e.g., pH ~7), FBA predictions would likely generate comparable results as a model generated by Thermo-Flux. However, in other conditions, mutants or non-model organisms, where no extensive curation has yet been performed, "Thermo-Flux" offers an easy way to generate a mechanistically correct model.

To address the reviewer's request to visualise the advances that can be achieved with models generated with Thermo-Flux, we added new analyses. Specifically, we predicted reaction directions using an *E. coli* model converted with Thermo-Flux. We constrained this thermodynamic model with physiological (https://doi.org/10.33612/diss.972628238) and metabolome (PMC5293157) data and performed thermodynamic flux variability analyses. We compared the predicted reaction directions against predefined reaction directions of an *E.*

coli stoichiometric model available in the BiGG database (iJR904). Here, we found several reaction directions that are different than the predefined reaction directions available in the stoichiometric model. We have added a new figure (Fig. 6b) to show these results. We also mention that mechanistically correct models are a requirement for improved predictions and included the two mentioned papers.

The automation of thermodynamic constraint application is a notable contribution, but it would be helpful for the authors to discuss why it is less automated than established protein resource constraint models. Given that thermodynamic constraints typically have lower data variability, the automation of this step could potentially be more robust and may benefit from further refinement.

Indeed, one would think that adding the second law of thermodynamics to metabolic reactions and assigning ΔrG° values to each of these reactions should not require much manual work. For most reactions, the assignment of ΔrG° values can indeed be automated. However, there are specific reactions that require extra attention and manual interventions. This includes reactions as the following ones:

1) reactions with metabolites for which no information is available on their structure or chemical formula or if a metabolite structure cannot be encoded in an InChi key (e.g., complex organometallics such as heme O). Here, the user needs to specify additional information that impact the estimation of the transformed Gibbs energy of formation, such as the number of binding protons and the charge of the metabolites. If there are redox cofactors whose formation energy cannot be estimated by *eQuilibrator* , the user needs to specify the Gibbs formation energies of the redox couple involved (see box 1).
2) transport processes with simultaneous chemical transformation prevent the automatic detection of transported metabolites performed in Thermo-Flux, as in such cases, no same metabolites appear on both sides of the membrane, and as such Thermo-Flux cannot identify the transported metabolite (see step 4).
3) transport processes that involve more than three compartments, as for the same reason than the previous point, Thermo-Flux cannot identify the transported species (mentioned in the methods).

Thus, there is a subset of special reactions and transport processes that require manual intervention. We have made this point clearer in the discussion of the revised manuscript.

It is unclear whether the Gibbs formation energies generated in Thermo-Flux are based on the same computational methods as those used in eQuilibrator. It would be beneficial for the authors to clarify this point and explain any differences between calculating metabolite formation energies with Thermo-Flux versus using eQuilibrator to generate parameters.

Thermo-Flux retrieves standard Gibbs formation energies of metabolites from eQuilibrator. Then, according to the model's physical and biochemical parameters (i.e. membrane potential

differences, compartmental pH values, ionic strength), Thermo-Flux transforms them using a Legendre transform, before calculating the Gibbs reaction energies. Finally, Thermo-Flux also calculates Gibbs reaction energies associated with the transport of charge and protons across membranes. We made this clearer in the revised version of the manuscript.

Biomass formation is treated as a metabolite with an associated Gibbs formation energy in the model. However, the authors should note that biomass formation is not a simultaneous process but rather a sequential one, meaning it does not strictly adhere to steady-state assumptions. Assigning a Gibbs formation energy to biomass formation may not be appropriate, and the synthesis of biomass could be excluded from thermodynamic constraints, as it does not fully align with the concept of simultaneous reactions and steady-state flux balancing.

We agree that biomass formation is a sequential process, at least in eukaryotes. According to what is common practice in the field of Flux Balance Analysis (FBA), we make the simplifying assumption of a simultaneous biomass formation process.

However, in line with the reviewer's suggestion, we do not constrain the biomass formation process with the 2nd law of thermodynamics. Still, we maintain the possibility to assign a Gibbs formation energy to biomass, as it is required for optimizations where a constraint on the upper limit of the Gibbs dissipation rate is used, as explained and discussed in Box 1. We made both points clearer in the revised manuscript.

Minor

Figure 1 could benefit from a clearer organization and a more logical flow of steps. A more intuitive layout would help readers better follow the described workflow.

We rearranged the layout to better match the figure with the described workflow.

The manuscript does not address the temperature dependency of the thermodynamic parameters. Since temperature can influence Gibbs energies, it would be useful to mention this limitation, particularly in the context of heat-tolerant organisms or industrial applications that may operate under extreme conditions.

This is a valid point. Temperature is indeed an important parameter for the thermodynamics of a biochemical reaction influencing the thermodynamics of a reaction in three different manners. In the equation, describing the Gibbs energy of a biochemical reaction, $\Delta_r G' = \Delta_r G'^{\circ} + RT \ln Q$, temperature affects:

1. the RT ln Q term
2. the standard transformed term $\Delta_r G'^{\circ}$ through enthalpy/entropy
3. the transform from chemical to biochemical standard state (Legendre transform), which also includes explicit RT terms and depends on acid/base and metal binding equilibria.

In the models generated by Thermo-Flux, the user provides a temperature as an input parameter. Thermo-Flux then considers point 1 and point 3 in its calculations of the transformed Gibbs formation energies. Point 2 is not considered at this point, the reason being that there is currently unfortunately insufficient knowledge on the enthalpies and entropies of biochemical reactions: The Gibbs energies from eQuilibrator were generated with experimental equilibrium constants measured in the mesophilic temperature range. A rough estimation of the enthalpy/entropy effects in the mesophilic temperature range shows that the changes in kJ/mol are within the uncertainties of the estimated Gibbs energies (doi: 10.1529/biophysj.107.124784). This means that, at least within the mesophilic temperature range, we do not expect any significant effects from omitting point 2.

However, and here the reviewer is right, moving to extremophiles, the picture would change. Then, enthalpies/entropies would need to be known such that one could apply respective correction. We now mention this limitation in the revised version of the manuscript.

Reviewer #2:

The authors present Thermo-flux, a software tool that automates the construction of Gibbs free energy-based thermodynamic constraints for genome-scale metabolic models (GEMs) and enables flux balance analysis (FBA) under those constraints. Thermodynamic constraints are known to reduce the feasible solution space and are expected to steer predictions toward biologically more meaningful states. However, assembling consistent free-energy bounds and implementing thermodynamics-constrained FBA (FBA,ST) is non-trivial. By addressing both tasks in a single workflow, Thermo-flux has the potential to broaden access to FBA,ST for routine users. In addition, FBA, ST automatically integrates metabolomic data with GEMs when such data is available. The study is timely and the software could be impactful. At the same time, in its current form it is difficult for me to assess the tool's practical usefulness and robustness. The manuscript would benefit from clearer methodological detail and stronger validation. Below are comments and suggestions to improve the paper.

Thanks a lot for the excellent summary of our work.

MAJOR COMMENTS

1) According to the Methods, it takes Thermo-flux FBA,S,T up to 24 hours to complete a single run with an average model:

"The calculations for (FBA - S,T) were run on the university's computational cluster 'Hábrók' and were optimized until the optimal solution was found, with a maximal duration of 24 h each."

If I understand correctly, this refers only to the mixed-integer linear optimization after all constraints are set. By comparison, parsimonious flux balance analysis (pFBA), which extends FBA by adding a secondary objective to minimize the total flux and thereby

removes futile cycles, typically solves in seconds on standard GEMs. While Thermo-flux offers more than just eliminating loops, a 24-hour solve time is a serious disadvantage relative to routine approaches. An alternative semi-standard method is loopless FBA.

My questions related to this issue are:

(1a) Is it true that FBA,S,T takes about 24 hours for the optimization step alone? Please clarify whether this is a typical runtime or a maximal wall-clock limit, and under what settings.

The mentioned 24 hours refer to a maximal wall-clock limit, which was hit when optimizing the largest models (i.e. the large-scale human model RECON1 with 3000+ reactions) where all reactions were set as reversible. Defining reaction directions using only the second law of thermodynamics (and not via *a priori* defined reaction directions) indeed slows down the computations. This slowing down is due to the fact that the number of possible combinations of reaction directions scales exponentially with the number of reactions. We agree that optimizing a model with fully reversible reaction directions is computationally intense, but we argue that such approach offer advantages in terms of mechanistic accuracy, as *a priori* defined reactions directions might be incorrect (as we now show in this revised version of the manuscript).

Smaller stoichiometric models take much less time (e.g., optimizing a yeast model with ~300 reactions is rather a question of minutes or single-digit hours), especially when constraints on exchange fluxes, for example based on physiological data, are added. We made this point clearer in the revised manuscript.

(1b) How long does Flux Variability Analysis (FVA) take under S,T constraints? Since FVA requires solving many optimizations (one or two per reaction), a full analysis with FBA,S,T and FVA,S,T could be impractically long; please provide typical runtimes.

FVA with thermodynamic constraints (FVA, S,T) optimizations with a 300-reaction model, in which all reactions are considered reversible, can take from a few minutes to several hours to optimize a single reaction, running on a few (1-4) cores, depending on the reaction that is optimized. To reduce the overall time for FVA optimizations, we typically up front exclude reactions that cannot carry flux under the respective condition from the set of reactions to be optimized, or first determine flux-coupled reactions (e.g. by flux coupling analysis on the stoichiometric models (DOI: 10.1186/s12918-015-0191-x; EFMTool or mparser) and then only FVA-optimize one of the “coupled” reactions. We revised the manuscript to cover this important point.

(1c) How does the optimization time compare with previously established loopless FBA algorithms on the same models and hardware?

Loopless FBA is faster (takes a few seconds) compared to FBA with thermodynamic constraints (takes several minutes to hours). However, while loopless FBA will not contain any

infeasible flux cycles, it is important to note that these solutions will not be constrained by the second law, relating metabolite concentrations and Gibbs reaction energies to fluxes. Reactions could still carry a flux even if metabolite concentrations and standard Gibbs reaction energies would imply a thermodynamically impossible reaction direction ($\Delta rG > 0$). Therefore, loopless FBA can still lead to biologically unrealistic solutions. In the revised version of the manuscript, we now provide runtimes comparisons of standard FBA (which runs in the same time as loopless FBA) and FBA with thermodynamic constraints.

2) The validations presented (Figure 6) are underwhelming, and it is not clear from these results whether Thermo-flux optimizations are superior or useful, or in what specific ways.

As the reviewer stated in the summary of our work at the beginning of this review, metabolic models with thermodynamics constraints reduce the solution space, in a mechanistic and accurate manner. This is a *necessary* but not *a sufficient* condition for improved predictions. We agree with the reviewer that it is interesting for our readers to know to what extent the predictions are better. We describe below the improvements we made in this regard in the revised version of the manuscript.

2a) The decrease in flux ranges from routine FBA to FBA,S,T in Fig. 6b appears minimal overall. Specific examples would be useful to demonstrate how FBA, ST is useful.

We agree with the reviewer that in this example, the flux range reduction was not huge. To give specific examples, as requested, we have added analyses to the result section, where we predicted reaction directions using a model converted with Thermo-Flux. We constrained this thermodynamic model with physiological (https://doi.org/10.33612/diss.972628238) and metabolome (PMC5293157) data and performed thermodynamic flux variability analyses. We compared the predicted reaction directions against predefined reaction directions of an *E. coli* stoichiometric model available in the BiGG database (iJR904).

In the revised version of the manuscript, we compare reaction directions instead of flux ranges because comparing fluxes and their ranges heavily depends on the model used for comparison. If one had a stoichiometric model with correctly defined reaction directions, the differences would likely not be so huge. Thus, the outcome of such a comparison depends on what one uses as a base model: we could compare flux predictions with a stoichiometric model using predefined directions, in which case our model would generate similar results. On the other hand, if we used a model with fewer predefined reaction directions, our model could generate better results. Therefore, we opted to analyze to what extent the reaction directions predicted with a thermodynamically constrained model and metabolome data would vary from the predefined ones.

Here, we found examples of reaction directions that are different than the predefined reaction directions as defined in the stoichiometric model (cf. Figure 6b), showing that these directions can be inconsistent with the second law and metabolome + physiological data. This provides motivation for why our work is important.

2b) The numbers in Fig. 6c report only how many changes occurred. Again, specific examples showing that the results change in a positive way would be useful.

As mentioned in the answer above, we now provide specific examples in the revised version of the manuscript.

2c) Fig. 6c, showing very accurate predictions of exchange fluxes, could be a strong validation, but it needs benchmarking against standard approaches: classical FBA, pFBA, and loopless FBA.

Following the request of the reviewer, we benchmarked predictions using standard approaches against predictions of exchange fluxes performed with a thermodynamically constrained model. First, we assessed whether FBA with thermodynamic constraints could accurately predict cellular physiology (e.g., extracellular fluxes) without relying on predefined reaction directions. To do so, we compared standard FBA using predefined reaction directions and FBA with thermodynamic constraints, in which reactions were initially fully reversible. We focused on low-glucose growth conditions, which do not exhibit overflow metabolism, because such phenotypes cannot be predicted without additional constraints such as an upper limit in the cellular Gibbs dissipation rate (https://doi.org/10.1007/s00018-019-03380-2). Here, we found that FBA with thermodynamic constraints predicted extracellular fluxes with an accuracy comparable to standard FBA with predefined reaction directions (Figure EV2). Thus, for low-glucose uptake conditions, thermodynamic constraints can be used to predict cellular physiology instead of relying on assumptions about reaction directionality, which, as seen above, may not always hold. We have added these results in the revised manuscript.

3) An important feature of Thermo-flux is the straightforward integration of metabolite concentrations when metabolomics data are available. This is not demonstrated by an example. It does not need to be network-scale. A small demonstration showing how narrowing metabolite concentration bounds affects ΔG'{degree sign}, reaction directionality, and flux ranges would be useful to illustrate the practical impact.

To demonstrate the impact of metabolome data as additional constraints, for this revised manuscript, we performed variability analyses using the *E. coli* model. First, we predicted reaction directions using thermodynamic constraints and exchange fluxes, constrained by physiological data. Then, we additionally constrained the model with experimental metabolome data. Here, we found several reactions that were still reversible without metabolome data but were constrained to a specific direction with metabolome data included (Fig. 7B). These results show that exploiting metabolome data in combination with thermodynamics can further constrain the flux solution space, compared to when only physiological data and a stoichiometric model is used. We added the results of these analyses to the revised manuscript.

MINOR COMMENTS (in the order of importance)

1) Page 8: addition of the artificial metabolite "charge" for charge balancing of compartments. It is not clear what this achieves. Transport reactions that only exchange ions like Na+ can balance out the "charge" metabolite in a straightforward manner with no mathematical effect on the solution. Please correct me if I am wrong and clarify the usefulness of this approach.

We agree that our previous text was not fully clear in this regard. The pseudo-metabolite “charge” has two roles. First, the pseudo-metabolite “charge” represents a positive charge and therefore could be used to model the transport of any generic cation, such as Na^+. Second, in metabolic networks, transport processes can be charge-neutral or involve charge movement. Movement of charges between compartments must be balanced to satisfy the steady state assumption and constant membrane potentials. Therefore, the pseudo-metabolite ‘charge’ is also used to store this information in the stoichiometric matrix and to track net charge transfer across membranes.

Moreover, charge movement affects the Gibbs reaction energy of a transport process, such that the feasibility of this reaction depends on the membrane potential difference between compartments. Therefore, a reaction that only moves a cation, such as Na^+, can only proceed following the membrane potential gradient. This means that if the flux required to maintain steady state is against the membrane potential gradient, such a reaction cannot contribute to balancing out the charge metabolite. Therefore, using thermodynamic constraints restricts the reactions that can contribute to charge balancing.

We have added these clarifications in the revised version of the manuscript.

2) Page 10: Equation 9. From the equation and its brief explanation, it appears to multiply precomputed constant free-energy values by fluxes, which would be linear (meaning the optimization problem would remain convex). I think the problem becomes non-convex due to mixed-integer variables as explained in the methods, but this should be clearly stated in the main text.

Equation 9 describes the second law of thermodynamics, relating the Gibbs reaction energy of a reaction to its flux. Note that equation 9 uses the Gibbs reaction energy $\Delta_r G$ and not the standard Gibbs reaction energy $\Delta_r G^{\circ}$. While $\Delta_r G^{\circ}$ is taken from eQuilibrator, $\Delta_r G$ depends on metabolite concentrations and an uncertainty term $\Delta_r G_{error}$, both of which are variables in the model. Because $\Delta_r G$ v can both vary during optimizations, this means that t$\Delta_r G \cdot v \leq 0$ is bilinear, rendering the solution space non-convex. The mixed-integer variables introduced in the Methods are used to model the conditionality of reaction directionality$\Delta_r G$. We have made this point clearer in the revised manuscript.

3) Page 10:"An upper limit in the cellular Gibbs energy dissipation rate" . It is never mentioned what the value of this limit is.

We agree that our previous text was not fully clear in this regard. In a previous study (Niebel et al., 2019), we had suggested that cells have a limit in the cellular Gibbs energy dissipation rate, which - when used as a constraint - led to improved predictions with a combined thermodynamic-stoichiometric model. We now explain this concept better in the revised version of the manuscript.

4) Page 14: "While it failed for a minority of models for reasons mentioned above, we have demonstrated with the iMM904 model that with limited additional manual effort, 'Thermo-Flux' can convert large stoichiometric models into combined thermodynamic stoichiometric models that enable improved predictions of metabolic fluxes." It is not clear what is meant by additional manual effort here.

We agree that the text was not clear enough. By "additional manual effort", we meant handling specific reactions that could not be automatically balanced using Thermo-Flux. For example, this concerns transport processes which include a chemical transformation step along the transport of a metabolite. We have included the lists of manual steps that we took to convert the iMM904 and the iJR904 models into thermodynamic-stoichiometric ones in the Supplementary tables S1 and S2.

5) Page 5, first sentence: "where NH is the number of hydrogen atoms in the species" I think what the authors mean is the number of hydrogen ions that can be gained or lost, not the total H atoms in a compound.

This is indeed a mistake. Thanks for spotting this. We have corrected it in the text.

6) Page 3: "pMg = 3.0," This is not consistent with the number in Table 1, which indicates 14

We agree that the text was not clear. Thermo-Flux uses a default pMg value of 3, when users do not specify any value. This is the value mentioned in the manuscript on page 3.

In contrast, the iMM904 case study uses a pMg value of 14 (as mentioned in Table 1). With such a high value (corresponding to an extremely low free Mg^{2+} concentration of 10^{-14} M), magnesium ions are effectively not considered in the model. This simplification means that no chemical species form complexes with magnesium, allowing us to simplify the case study by avoiding magnesium balancing in the reactions and therefore reducing the number of distinct biochemical species that are considered in the balancing.

Therefore, the inconsistency noted by the reviewer is due to page 3 reporting the default setting, while Table 1 reports the parameter choice for the iMM904 model case study. We made this clearer in the revised version of the manuscript.

7) Box 1: Please explain when photon energy can be useful in metabolic modeling.

The photosystems of plants and other organisms, relying on photosynthesis, is an important part of their metabolism, as these organisms use photons from light to generate ATP and NADPH. Thus, when a stoichiometric model of a plant is converted into a thermodynamic-stoichiometric model, the energetics of photons need to be included in the model. Published stoichiometric models that include photons as metabolites exist, for instance, for cyanobacteria (e.g., iJN678 and iSynCJ816 in BiGG) and algae (e.g., iLB1027_lipid in BiGG). We added a brief explanation as to why photon energy is important when modeling organisms with photosynthesis.

Reviewer #3:

The authors present Thermo-Flux, an automated pipeline to reconstruct thermodynamically constrained stoichiometric models. The pipeline is designed to constrain reaction directionalities in stoichiometric models (e.g., genome-scale metabolic models) according to the second law of thermodynamics. While similar tools exist for this purpose, this pipeline in particular offers automated charge/proton balancing and the calculation of thermodynamic properties for transport reactions and uncommon reactions such as growth. The reviewer agrees that presenting a fully automated pipeline, compatible with recent advances in machine learning and data science to account for thermodynamics in metabolic models, is significant and potentially impactful. However, the current pipeline proposed by the authors has important shortcomings and does not offer a substantial improvement over previously published methods.

Major comments

1. One of the main contributions of this paper is the proposal of an automated pipeline for charge/proton balancing of reactions. However, the method presented by the authors is confusing and results in reactions with non-integer stoichiometric coefficients. Even if one assumes that users do not need to fully understand the underlying balancing mechanism and only need to apply the tool, they must ultimately be able to interpret the resulting reactions. When users inspect the final model, they should have a clear sense of what these reactions represent, which is not the case with the current formulation.

The automated charge and proton-balancing is indeed one of the important contributions of our work. This aspect is novel because current metabolic network models usually ignore the fact that biochemical reactants, such as ATP, exist as different chemical species. Yet, ignoring the simultaneous presence of species of various protonation states for one single reactant can lead to representing and modeling an incorrect number of protons binding to that reactant. Failing to properly account for the number of protons involved in the overall reaction impacts (i) the mass balance enforcement, because the net production/consumption of protons will be inaccurate, and (ii) the calculation of the Gibbs reaction energy of transport

processes, because of the term, which considers the movement of charge or protons across the membrane (cf. step 6 of the manuscript).

The thermodynamic-stoichiometric models as generated with Thermo-Flux consider the fact that reactants are present as different species, which inevitably leads to non-integer stoichiometric coefficients for protons in these models. We realize that this is a novel aspect, which we needed to explain better such that readers fully understand this point. In this revised version, we made this point clearer. To this end, the next comment made by this reviewer helped us finding a good way to explain the occurrence of non-integer numbers to our readers. Thanks a lot for bringing this point up!

2. Along the same lines, I propose the following alternative approach to reaction balancing. Starting from the example presented in the paper, let us assume a reaction involving phosphate as one of the reactants, and that phosphate exists in two forms at the given pH, namely PO_4^{3-} and HPO_4^{2-}. Instead of the formulation presented by the authors, the original reaction could be rewritten as the following set of reactions:

a. $PO_4^{3-} + A \rightarrow B$

b. $HPO_4^{2-} + A \rightarrow B'$

c. $H^+ + PO_4^{3-} \leftrightarrow HPO_4^{2-}$

Assuming a constant steady-state pH, all reactions are mass- and charge-balanced. Moreover, if one wishes to enforce a specific ratio between the two phosphate species, similar to what the authors aim to achieve, this can be done through constraints on metabolite concentrations. This formulation is easier to interpret and does not require the additional assumptions and complexity introduced by the authors' approach.

In principle, this would be an approach that would be a bit easier to follow. However, adopting this approach would mean that each metabolite would need to be turned into several species, which would significantly increase the number of reactions, which likely will have a negative impact on the computation times. In the light of the already significant computational times required for these models (see comments of reviewer 2), we would prefer to stick with the approach that uses non-integer stoichiometric comments.

However, to address this reviewer’s point, in the revised version of the manuscript, we carefully explain this aspect to our readers: first, we explain that, in principle, we would have to include the balances for all species (similar to the example given the reviewer here). We then mention that this would significantly increase the number of reactions and thus we merge the reactions of the different species of a reactant into one single reaction, which leads to non-integer numbers. We hope that this explanation, added to the revised version of the manuscript, will clarify for readers why the thermodynamic-stoichiometric models generated by Thermo-Flux can have non-integer stoichiometric coefficients.

3. The authors mention differences in metabolite concentrations across cellular compartments. This is an important consideration, but it was not clear whether or how this was incorporated into their formulation. The authors should clarify this point.

We understand the confusion, as this point was indeed not explained well. In our approach, we define compartment-specific metabolite concentrations by introducing separate concentration variables for each compartment. For example, a metabolite A located in the cytosol and in the mitochondria is represented by two distinct concentration variables, A_{cyt} and A_{mit}. These variables are treated independently in the model. If metabolome data are available and a user wants to add such data as constraints, we relate the whole-cell metabolite concentrations to the compartment-specific concentrations via simple algebraic equations weighted by compartmental volume fractions (cf. Box 2). We have clarified this point in the revised manuscript.

4. Page 12, second paragraph of the Results section, should be improved. Rather than stating the obvious, the authors should analyze these results in more depth. Which reactions were over-constrained? What were the underlying reasons (e.g., errors in Gibbs energy estimation, incorrect balancing, uncertainty in reaction participants)? Are there insights from these cases that could be used to improve the proposed pipeline?

To address this comment, we now investigated why some models became infeasible after adding thermodynamic constraints. We identified two main causes of infeasibility. First, infeasibility arose from incorrect reaction stoichiometries in transport processes, even after the automated proton balancing done in 'Thermo-Flux', which resulted in an incorrect number of protons being available for transport compared to what was required to maintain a steady-state. This issue requires manual curation of transport processes, as demonstrated in the iMM904 case study, to ensure that the movement of protons is modeled accurately and that the resulting Gibbs reaction energies of the transport processes are accurate. Now that we identified that such errors could exist in stoichiometric models, we added a new function in Thermo-Flux to allow users to easily add proton transport reactions between compartments.

Second, certain downloaded models contained biomass reactions with stoichiometric coefficients in the order of thousands (e.g., 35 115 ATP per biomass produced in iSB619), indicating that the biomass equation had not been scaled to produce 1 g of biomass. Such large stoichiometric coefficients scale $\Delta_r G°$ values proportionally, yielding values so extreme that no feasible combination of metabolite concentrations and Gibbs energy uncertainties could satisfy the thermodynamic constraints, rendering the optimization infeasible.

We have revised the manuscript to include these additional insights.

Minor comments

1. In Box 2, the authors state that for paired metabolites lacking an explicit biosynthesis pathway (e.g., NAD(P)/NAD(P)H), they set the concentration of one cofactor to 1 M. What do the authors mean by "explicit biosynthesis pathway"? Organisms such as E. coli or yeast can synthesize these compounds from simple carbon sources like glucose. Moreover, if these compounds participate in other reactions, setting their concentration to such a high value could introduce inconsistencies or artifacts in the overall simulations.

Thanks for bringing this up. We indeed did not explained this properly. Predictions with thermodynamic-stoichiometric models benefit from bounds on metabolite concentrations. The tighter the bounds are, the more constrained the model will be and the greater the chance of improved predictions. While for "normal" metabolites, concentration bounds are needed for each metabolite, for redox cofactors, ratios of concentrations are sufficient to constrain the thermodynamics of the reaction (because the oxidized/reduced cofactor pair appears on either side of the reaction). Using redox concentration ratios is advantageous in this case because it is inherently difficult to measure the *concentrations* of redox cofactors, whereas estimates of their *ratios* are readily available, even in a compartment-resolved manner (PMID: 26059529).

To apply a constraint on the redox cofactor ratio, we set one cofactor to 1 M. By fixing one cofactor at 1 M, its contribution to the reaction energy equation becomes zero (since ln(1) = 0), reducing the two concentration variables to the concentration ratio of the cofactors. For example, for the NAD^+/NADH pair, fixing the concentration of NAD+ to 1 M ensures that the concentration of NADH directly represents the NADH/NAD^+ concentrations ratio in all thermodynamic calculations.

It is important to note that this approach can only be followed for metabolites that do not contain de novo synthesis or salvage reactions in the model (such as Ferrocytochrome/Ferricytochrome in the iMM904 model), because the concentration of the synthesized or salvaged metabolite appears individually (i.e. not as pair) in such reactions, as the reviewer correctly states here. We have made this point clearer in the manuscript.

2. In my experience, the impact of integrating thermodynamic constraints is particularly evident for reactions involving novel compounds (e.g., xenobiotics), as the directionality of native and common reactions is usually well curated. Can this pipeline be applied to reactions involving novel compounds, especially those not present in the eQuilibrator database? The authors should comment on this point and discuss the potential requirements or limitations of their approach in such cases.

eQuilibrator is already pretty good in estimating ΔrG values for reactions whose Gibbs reaction energies have not been measured experimentally (cf. https://doi.org/10.1371/journal.pcbi.1003098). This feature of eQuilibrator already gets us quite far. However, there are cases where eQuilibrator cannot return a Gibbs formation

energy for a compound, for instance, if its structure is unknown or cannot be decomposed by the component contribution method. As explained in box 1 of our manuscript, for such compounds, the uncertainty of the Gibbs formation energy will be high, but still this uncertainty is considered in Thermo-Flux's thermodynamic constraints. In cases, where a reaction has several metabolites with unknown Gibbs formation energy, the respective ΔrG° can span a wide range, such that the ΔrG of the reaction can eventually still take any positive or negative value. Therefore, a possible limitation when reactions involve novel compounds is that such reactions can take any direction even under the thermodynamic constraints. We now mention this point in the manuscript.

3. In some eukaryotic cells, for example yeast-which the authors use as a demonstration case-metabolism occurs in multiple membranous compartments. Most equations used to calculate thermodynamic properties are developed for aqueous conditions, whereas membrane environments do not readily satisfy these assumptions. Did the authors make any special considerations for reactions occurring in or across membranes?

In the yeast model used in our manuscript, reactions are assigned to different compartments. For reactions confined to a single compartment, Gibbs energies are calculated using compartment-specific pH and ionic strength values consistent with published yeast physiology. For transport reactions that move species across compartments, Thermo-Flux accounts for differences in pH, ionic strength, and charge between the respective compartments to yield thermodynamically consistent ΔrG° values for each transport process. So, this point is well taken care of.

However, we acknowledge that reactions occurring within the lipid bilayer (such as those catalysed by membrane-embedded enzymes, such as those of the respiratory chain complexes) represent a limitation of the current framework. This limitation manifests in two ways: First, ΔfG° estimates for membrane-phase species are unreliable, as group contribution methods are trained on aqueous-phase data and perform poorly for compounds with long hydrophobic tails; and second, the concentration-dependent term of the Gibbs energy is ill-defined for species lacking a true aqueous concentration. To mitigate over-constraining reaction directionality with inaccurate thermodynamic data, we recommend assigning wide concentration bounds to such species, a practice also suggested by the eQuilibrator community (cf. https://groups.google.com/g/equilibrator-users/c/ARvwhQSo5rU/m/XOwFTdbIAQAJ). The practical impact of this limitation, however, is expected to be low, as the affected reactions - primarily those in the electron transport chain - operate with a large thermodynamic driving force and a well-established directionality, meaning the thermodynamic constraint is effectively non-binding in practice.

This is a recognized limitation in the field (https://doi.org/10.1038/s41467-021-25158-6 ; https://doi.org/10.1002/bit.260361013), and to our knowledge no genome-scale modelling approach using thermodynamics currently accounts for the non-aqueous condition of

membrane compartments. We now mention this limitation in the revised discussion section of the manuscript and have added the mentioned recommendation in the documentation of the package.

1st Jun 2026

Manuscript Number: MSB-2025-13491R
Title: Thermo-Flux: generation and analysis of comprehensive thermodynamic-stoichiometric metabolic network models
Author: Edward Smith
Nathan Fargier
José Pedro
Matthias Heinemann

Dear Matthias,

Thank you for submitting the revised version of your manuscript. We have now received feedback from the three reviewers who were asked to re-evaluate your study. As you will see from the comments below, Reviewers #1 and #2 are satisfied with the revisions, while Reviewer #3 still raises some concerns about the depth of the analysis.

Taking all three reports into consideration, and given that Reviewer #3's remaining concerns are not related to specific technical issues, we have decided to invite a minor revision.

Before we can formally accept your manuscript for publication, we ask that you address the remaining minor points raised by Reviewer #2.

Please also note that Reviewer #2 provided additional comments on your responses to the reviewers' previous concerns. In the interest of transparency, I have included these remarks below, following their review report.

On a more editorial level :

1. Please provide a Word version of the manuscript that excludes all figures, the Synopsis text, and the graphical abstract. In addition, footnotes listing the corresponding author should be placed directly below the author list on the title page.

2. Please provide each main and EV figure as a separate, publication-quality file in one of the following formats: .eps, .tif, or .jpg (one file per figure). Their legends should remain in the manuscript file, placed after the References section.

3. The three EV tables should be uploaded as separate files, with one file per table. Each file should include a separate sheet labeled 'Legend' containing the corresponding table legend.

4. The Appendix should be removed from the main manuscript file and uploaded as a separate PDF document named "Appendix." All appendix items should be numbered using the nomenclature "Appendix Table Sx". The first page of the Appendix must contain a Table of Contents listing all the listed items, together with their corresponding page numbers. The title page should be headed: Appendix for "[Manuscript Title]"

5. Please add missing callouts for Fig. 4B, 5A, 5B.

6. Please provide the visual abstract as a separate PNG file with dimensions of exactly 550 px (width) × 300 px (height).

7. Please also provide the Standfirst and Bullet Points as a separate Word document, uploaded independently from the main manuscript files.

8. The references need to be formatted according to the Molecular Systems Biology reference style. Please list up to 10 co-authors of a paper before adding et al. in the reference list. Citations should be listed in alphabetical order.

9. "Disclosure statement and competing interests" should be renamed to "Disclosure and competing interests statement" .

10. Sections need to be named and the order should be corrected: Title page - Abstract - Introduction - Results - Discussion - Methods - Data Availability - Acknowledgements - Disclosure and Competing Interests Statement - References - Figure Legends - Table(s) - Expanded View Figure Legends.

Use the following link to submit your revised paper:

https://msb.msubmit.net/cgi-bin/main.plex

As a matter of course, please make sure that you have correctly followed the instructions for authors as given on the submission website.

Thank you for submitting this paper to Molecular Systems Biology.

Kind regards,
Jingyi

Jingyi Hou, PhD
Senior Editor
Molecular Systems Biology

*** PLEASE NOTE *** As part of the EMBO Press transparent editorial process initiative (see our Editorial at https://dx.doi.org/10.1038/msb.2010.72 , Molecular Systems Biology will publish online a Review Process File to accompany accepted manuscripts. When preparing your letter of response, please be aware that in the event of acceptance, your cover letter/point-by-point document will be included as part of this File, which will be available to the scientific community. More information about this initiative is available in our Instructions to Authors. If you have any questions about this initiative, please contact the editorial office (msb@embo.org).

Reviewer #1:

This work makes a significant contribution to model construction by addressing key challenges, including the thermodynamics of transport processes and the treatment of metabolites with unmeasurable energies. It provides a semi-automated workflow for parameter prediction steps that previously required extensive manual effort and impacted prediction accuracy. Given the substantial improvements made by the authors, I support the approaches and conclusions presented in the current manuscript and recommend acceptance, enabling timely reference and application of this work by the community.

Reviewer #2:

The authors addressed all of my concerns. I think the publication of this work would be a useful step forward for those of us who are interested in systematically trying thermodynamic modeling, but hesitating because of current limitations and a steep "activation energy barrier", which would be reduced by this work.

Minor comments
*Table S1: must be Table EV1 according to text.

*Table S1: Why doesn't yeast have vacuole? If this is due to the yeast GEM's limitations, it should be indicated as a foot note.

*Text related to Equation 2: "I" must be "IS" for Ionic strength

*Fig 6B: Blocked boxes do not seem to exist, so please remove this description from the legend. Also, why are ACODA and GLCpts included in this comparison, if they do not change reversibility between different models?

Assessment of Responses to Other Reviewers

Reviewer 1 major comment 1
It appears that the suggested benchmarking against given reference models have not been done; these references are only discussed in the paper. One potential reason for lack of further action is that the models suggested by the reviewer do not provide an automated (or semi-automated) model building procedure, and a low number of samples for comparison would not useful. If true, I would agree with the authors' position.

Reviewer 3, comment 1: I do NOT agree (with the reviewer) that inclusion of non-integer charges is a serious concern. A metabolite in a GEM can even have non-integer formulas (e.g. C16.3O...) reflecting a composition rather than a single form (such as in a proper metabolite representation of total phosphatidyl choline with an ambiguous R group in a classical formula limited to integers). These should not be confusing for modelers.

Reviewer #3:

I would like to thank the authors for their efforts in addressing my previous comments. While several of my concerns have been clarified, I believe the manuscript could still be further strengthened, particularly regarding the depth of the analysis and evaluation presented. Overall, I recognize the potential value of the work; however, in its current form, I do not feel able to support publication at this stage. Nevertheless, I leave the final assessment to the editor and the other reviewers.

Response to minor comments from reviewer 2 :

Minor comment: *Table S1: must be Table EV1 according to text.*

Modifications:
We thank the reviewer for pointing out the inconsistency. We have corrected the references throughout the manuscript where Table EV1 and Table S1 were mixed up. Table EV1 shows example physical and biochemical parameters, whereas Table S1 contains code snippets illustrating the protocol steps using the conversion of the iMM904 model as a case study.

Minor comment: *Table S1: Why doesn't yeast have vacuole? If this is due to the yeast GEM's limitations, it should be indicated as a foot note.*

Modifications:
We thank the reviewer for this observation. We believe the reviewer is referring to Table EV1, which lists example parameter values used in the model. The iMM904 model, which we converted into a combined thermodynamic-stoichoimetric model, does include a vacuolar compartment, and a vacuolar pH of 5.5 was assigned (Martino Mullez et al. 2008). However, this value was omitted from Table EV1. We have now added this entry to Table EV1.

Minor comment: *Text related to Equation 2: "I" must be "IS" for Ionic strength.*

Modifications:
We thank the reviewer for spotting this inconsistency. Because ionic strength is represented by the symbol *I* throughout the Thermo-Flux code, but Equation 2 was using 'IS', we have modified Equation 2 to use *I* instead of *IS*. This change ensures consistent notation for ionic strength throughout the manuscript and Thermo-Flux.

Minor comment: *Fig 6B: Blocked boxes do not seem to exist, so please remove this description from the legend. Also, why are ACODA and GLCpts included in this comparison, if they do not change reversibility between different models?*

Modifications:
We thank the reviewer for spotting this mistake. We have updated the reactions displayed in the fig 6B, to (i) display a reaction that has a predefined direction set to forward and is predicted to be blocked in the VA, thereby matching the description provided in the figure legend, and (ii) to replace ACODA (Acetylornithine deacetylase) and GLCpts by ACOTA (acetylornithine transaminase) and A5PISO. Both ACOTA and A5PISO change direction/reversibility between the predefined directions and the variability analyses results, making them more appropriate examples for this comparison.

10th Jun 2026
Manuscript number: MSB-2025-13491RR
Title: Thermo-Flux: generation and analysis of thermodynamic-stoichiometric metabolic network models

Dear Matthias,

Thank you again for sending us your revised manuscript. We are now satisfied with the modifications made and I am pleased to inform you that your paper has been accepted for publication.

Your manuscript will be processed for publication by EMBO Press. It will be copy edited and you will receive page proofs prior to publication. Please note that you will be contacted by Springer Nature Author Services to complete licensing and payment information.

You may qualify for financial assistance for your publication charges - either via a Springer Nature fully open access agreement or an EMBO initiative. Check your eligibility: https://link.springer.com/journal/44320/how-to-publish-with-us

Should you be planning a Press Release on your article, please notify the production team via this form in order to coordinate publication and release dates: https://forms.cloud.microsoft/e/eH4A8JVP6g

If you have any questions, please do not hesitate to contact the Editorial Office. Thank you for your contribution to Molecular Systems Biology.

Sincerely,
Jingyi

Jingyi Hou, PhD
Senior Editor
Molecular Systems Biology

>>> Please note that it is Molecular Systems Biology policy for the transcript of the editorial process (containing referee reports and your response letter) to be published as an online supplement to each paper. If you do NOT want this, you will need to inform the Editorial Office via email immediately. More information is available here: https://link.springer.com/partners/embo-press/editorial-policies#Peer%20review

EMBO Press Author Checklist

Corresponding Author Name: Matthias Heinemann	
Journal Submitted to: Molecular Systems Biology	
Manuscript Number: MSB-2025-13491	

USEFUL LINKS FOR COMPLETING THIS FORM
The EMBO Journal - Author Guidelines
EMBO Reports - Author Guidelines
Molecular Systems Biology - Author Guidelines
EMBO Molecular Medicine - Author Guidelines

Reporting Checklist for Life Science Articles (updated January 2022)

This checklist is adapted from Materials Design Analysis Reporting (MDAR) Checklist for Authors. MDAR establishes a minimum set of requirements in transparent reporting in the life sciences (see Statement of Task: 10.31222/osf.io/9sm4x). Please follow the journal's guidelines in preparing your manuscript.
Please note that a copy of this checklist will be published alongside your article.

Abridged guidelines for figures
1. Data
The data shown in figures should satisfy the following conditions:
- the data were obtained and processed according to the field's best practice and are presented to reflect the results of the experiments in an accurate and unbiased manner.
- ideally, figure panels should include only measurements that are directly comparable to each other and obtained with the same assay.
- plots include clearly labeled error bars for independent experiments and sample sizes. Unless justified, error bars should not be shown for technical replicates.
- if n<5, the individual data points from each experiment should be plotted. Any statistical test employed should be justified.
- Source Data should be included to report the data underlying figures according to the guidelines set out in the authorship guidelines on Data Presentation.

2. Captions
Each figure caption should contain the following information, for each panel where they are relevant:
- a specification of the experimental system investigated (eg cell line, species name).
- the assay(s) and method(s) used to carry out the reported observations and measurements.
- an explicit mention of the biological and chemical entity(ies) that are being measured.
- an explicit mention of the biological and chemical entity(ies) that are altered/varied/perturbed in a controlled manner.
- the exact sample size (n) for each experimental group/condition, given as a number, not a range;
- a description of the sample collection allowing the reader to understand whether the samples represent technical or biological replicates (including how many animals, litters, cultures, etc.).
- a statement of how many times the experiment shown was independently replicated in the laboratory.
- definitions of statistical methods and measures:
  - common tests, such as t-test (please specify whether paired vs. unpaired), simple χ2 tests, Wilcoxon and Mann-Whitney tests, can be unambiguously identified by name only, but more complex techniques should be described in the methods section;
  - are tests one-sided or two-sided?
  - are there adjustments for multiple comparisons?
  - exact statistical test results, e.g., P values = x but not P values < x;
  - definition of 'center values' as median or average;
  - definition of error bars as s.d. or s.e.m.

Please complete ALL of the questions below.
Select "Not Applicable" only when the requested information is not relevant for your study.

Materials

Newly Created Materials	Information included in the manuscript?	In which section is the information available? (Reagents and Tools Table, Materials and Methods, Figures, Data Availability Section)
New materials and reagents need to be available; do any restrictions apply?	Not Applicable	

Antibodies	Information included in the manuscript?	In which section is the information available? (Reagents and Tools Table, Materials and Methods, Figures, Data Availability Section)
For **antibodies** provide the following information:<br>- Commercial antibodies: RRID (if possible) or supplier name, catalogue number and or/clone number<br>- Non-commercial: RRID or citation	Not Applicable	

DNA and RNA sequences	Information included in the manuscript?	In which section is the information available? (Reagents and Tools Table, Materials and Methods, Figures, Data Availability Section)
**Short novel DNA or RNA including primers, probes**: provide the sequences.	Not Applicable	

Cell materials	Information included in the manuscript?	In which section is the information available? (Reagents and Tools Table, Materials and Methods, Figures, Data Availability Section)
**Cell lines:** Provide species information, strain. Provide accession number in repository **OR** supplier name, catalog number, clone number, and/**OR** RRID.	Not Applicable	
**Primary cultures:** Provide species, strain, sex of origin, genetic modification status.	Not Applicable	
Report if the cell lines were recently **authenticated** (e.g., by STR profiling) and tested for mycoplasma contamination.	Not Applicable	

Experimental animals	Information included in the manuscript?	In which section is the information available? (Reagents and Tools Table, Materials and Methods, Figures, Data Availability Section)
**Laboratory animals or Model organisms:** Provide species, strain, sex, age, genetic modification status. Provide accession number in repository **OR** supplier name, catalog number, clone number, **OR** RRID.	Not Applicable	
**Animal observed in or captured from the field:** Provide species, sex, and age where possible.	Not Applicable	
Please detail **housing and husbandry conditions**.	Not Applicable	

Plants and microbes	Information included in the manuscript?	In which section is the information available? (Reagents and Tools Table, Materials and Methods, Figures, Data Availability Section)
**Plants:** provide species and strain, ecotype and cultivar where relevant, unique accession number if available, and source (including location for collected wild specimens).	Not Applicable	
**Microbes:** provide species and strain, unique accession number if available, and source.	Not Applicable	

Human research participants	Information included in the manuscript?	In which section is the information available? (Reagents and Tools Table, Materials and Methods, Figures, Data Availability Section)
If collected and within the bounds of privacy constraints report on age, sex and gender or ethnicity for all study participants.	Not Applicable	

Core facilities	Information included in the manuscript?	In which section is the information available? (Reagents and Tools Table, Materials and Methods, Figures, Data Availability Section)
If your work benefited from core facilities, was their service mentioned in the acknowledgments section?	Not Applicable	

Design

Study protocol	Information included in the manuscript?	In which section is the information available? (Reagents and Tools Table, Materials and Methods, Figures, Data Availability Section)
If study protocol has been **pre-registered, provide DOI in the manuscript**. For clinical trials, provide the trial registration number **OR** cite DOI.	Not Applicable	
Report the **clinical trial registration number** (at ClinicalTrials.gov or equivalent), where applicable.	Not Applicable	

Laboratory protocol	Information included in the manuscript?	In which section is the information available? (Reagents and Tools Table, Materials and Methods, Figures, Data Availability Section)
Provide DOI OR other citation details if **external detailed step-by-step protocols** are available.	Not Applicable	

Experimental study design and statistics	Information included in the manuscript?	In which section is the information available? (Reagents and Tools Table, Materials and Methods, Figures, Data Availability Section)
Include a statement about **sample size** estimate even if no statistical methods were used.	Not Applicable	
Were any steps taken to minimize the effects of subjective bias when allocating animals/samples to treatment (e.g. **randomization procedure**)? If yes, have they been described?	Not Applicable	
Include a statement about **blinding** even if no blinding was done.	Not Applicable	
Describe **inclusion/exclusion criteria** if samples or animals were excluded from the analysis. Were the criteria pre-established?<br><br>If sample or data points were omitted from analysis, report if this was due to attrition or intentional exclusion and provide justification.	Not Applicable	
For every figure, are **statistical tests** justified as appropriate? Do the data meet the assumptions of the tests (e.g., normal distribution)? Describe any methods used to assess it. Is there an estimate of variation within each group of data? Is the variance similar between the groups that are being statistically compared?	Not Applicable	

Sample definition and in-laboratory replication	Information included in the manuscript?	In which section is the information available? (Reagents and Tools Table, Materials and Methods, Figures, Data Availability Section)
In the figure legends: state number of times the experiment was **replicated** in laboratory.	Not Applicable	
In the figure legends: define whether data describe **technical or biological replicates**.	Not Applicable	

Ethics

Ethics	Information included in the manuscript?	In which section is the information available? (Reagents and Tools Table, Materials and Methods, Figures, Data Availability Section)
Studies involving **human participants**: State details of **authority granting ethics approval** (IRB or equivalent committee(s), provide reference number for approval.	Not Applicable	
Studies involving **human participants**: Include a statement confirming that **informed consent** was obtained from all subjects and that the experiments conformed to the principles set out in the WMA Declaration of Helsinki and the Department of Health and Human Services Belmont Report.	Not Applicable	
Studies involving **human participants:** For publication of **patient photos**, include a statement confirming that consent to publish was obtained.	Not Applicable	
Studies involving experimental **animals**: State details of **authority granting ethics approval** (IRB or equivalent committee(s), provide reference number for approval. Include a statement of compliance with ethical regulations.	Not Applicable	
Studies involving **specimen and field samples:** State if relevant **permits** obtained, provide details of authority approving study; if none were required, explain why.	Not Applicable	

Dual Use Research of Concern (DURC)	Information included in the manuscript?	In which section is the information available? (Reagents and Tools Table, Materials and Methods, Figures, Data Availability Section)
Could your study fall under dual use research restrictions? Please check biosecurity documents and list of select agents and toxins (CDC): https://www.selectagents.gov/sat/list.htm	Not Applicable	
If you used a select agent, is the security level of the lab appropriate and reported in the manuscript?	Not Applicable	
If a study is subject to dual use research of concern regulations, is the name of the **authority granting approval and reference number** for the regulatory approval provided in the manuscript?	Not Applicable	

Reporting

The MDAR framework recommends adoption of discipline-specific guidelines, established and endorsed through community initiatives. Journals have their own policy about requiring specific guidelines and recommendations to complement MDAR.

Adherence to community standards	Information included in the manuscript?	In which section is the information available? (Reagents and Tools Table, Materials and Methods, Figures, Data Availability Section)
State if relevant guidelines or checklists (e.g., **ICMJE, MIBBI, ARRIVE, PRISMA**) have been followed or provided.	Not Applicable	
For **tumor marker prognostic studies,** we recommend that you follow the **REMARK** reporting guidelines (see link list at top right). See author guidelines, under 'Reporting Guidelines'. Please confirm you have followed these guidelines.	Not Applicable	
For **phase II and III randomized controlled trials**, please refer to the **CONSORT** flow diagram (see link list at top right) and submit the CONSORT checklist (see link list at top right) with your submission. See author guidelines, under 'Reporting Guidelines'. Please confirm you have submitted this list.	Not Applicable	

Data Availability

Data availability	Information included in the manuscript?	In which section is the information available? (Reagents and Tools Table, Materials and Methods, Figures, Data Availability Section)
Have **primary datasets** been deposited according to the journal's guidelines (see 'Data Deposition' section) and the respective accession numbers provided in the Data Availability Section?	Not Applicable	
Were **human clinical and genomic datasets** deposited in a public access-controlled repository in accordance to ethical obligations to the patients and to the applicable consent agreement?	Not Applicable	
Are **computational models** that are central and integral to a study available without restrictions in a machine-readable form? Were the relevant accession numbers or links provided?	Yes	The Github link can be found in the Data availability section.
If publicly available data were reused, provide the respective **data citations in the reference list**.	Not Applicable	
