# Supplementary material for: Thermo-flux: generation and analysis of thermodynamic-stoichiometric metabolic network models
Source: Mol Syst Biol. 2026 Jul 3;22(10):1650–64. doi: 10.1038/s44320-026-00227-4 (PMC13639002; doi:10.1038/s44320-026-00227-4)
Supplement: Supplementary file 3 [file 44320_2026_227_MOESM6_ESM.pdf]

Expanded View Figures

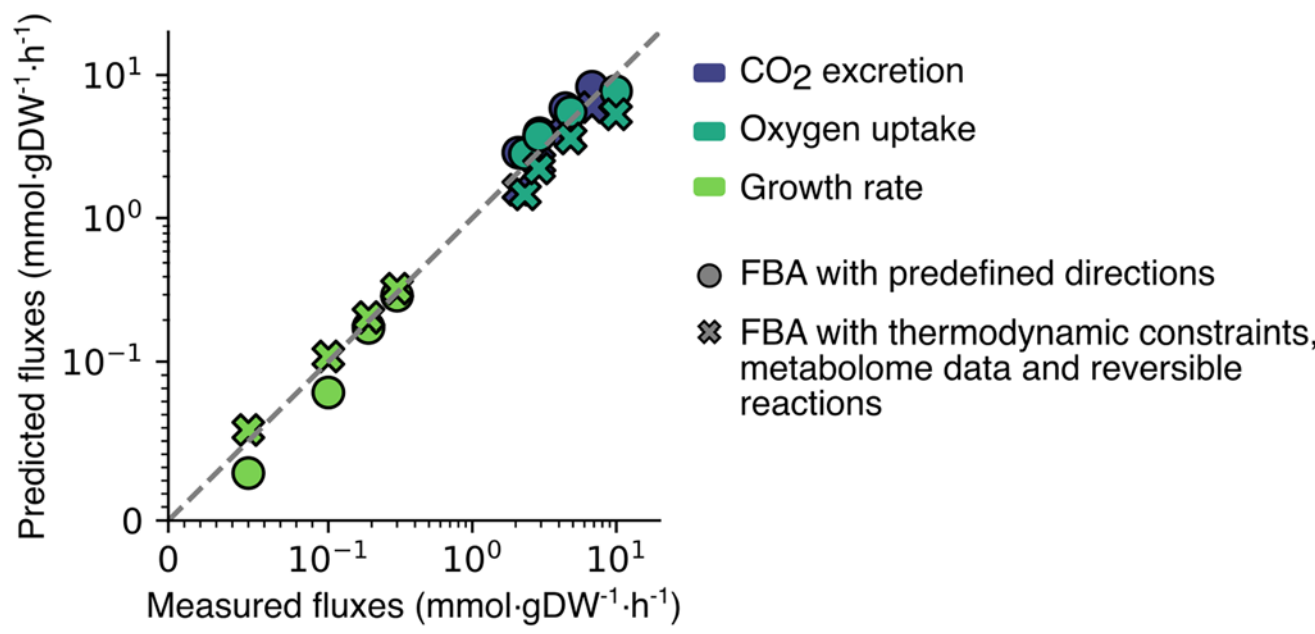

Figure EV1. Comparison of predicted and experimental extracellular fluxes in *E. coli* at four low-glucose uptake rates.

The fluxes were predicted using either (i) FBA with the iJR904 stoichiometric model with its predefined reaction directions, or (ii) FBA with the *E. coli* model built with "Thermo-Flux", which incorporates thermodynamic constraints and metabolome data as bounds on measured metabolite concentrations, but fully reversible reactions. With the iJR904 model, loopless FBA and pFBA predicted the same results as standard FBA in terms of exchange fluxes, these predictions are not included in this figure. All FBA optimizations maximized biomass production. The experimental values were measured in chemostat cultures (Vemuri et al, 2007) and were measured only for CO_2 excretion, O_2 uptake and growth rate.

© The Author(s)
